# Glutamate dynamics in the dorsolateral striatum of rats with goal-directed and habitual cocaine-seeking behavior

**DOI:** 10.3389/fnmol.2023.1160157

**Published:** 2023-05-11

**Authors:** Danielle M. Giangrasso, Kaliana M. Veros, Maureen M. Timm, Peter J. West, Karen S. Wilcox, Kristen A. Keefe

**Affiliations:** ^1^Department of Pharmacology & Toxicology, University of Utah, Salt Lake City, UT, United States; ^2^Interdepartmental Program in Neuroscience, University of Utah, Salt Lake City, UT, United States; ^3^Anticonvulsant Drug Development Program, Department of Pharmacology & Toxicology, University of Utah, Salt Lake City, UT, United States

**Keywords:** dorsal striatum, cocaine, habitual and goal-directed processes, devaluation, astrocytes, glutamate transporter, synaptic transport currents, iGluSnFr

## Abstract

The shift from drug abuse to addiction is considered to arise from the transition between goal-directed and habitual control over drug behavior. Habitual responding for appetitive and skill-based behaviors is mediated by potentiated glutamate signaling in the dorsolateral striatum (DLS), but the state of the DLS glutamate system in the context of habitual drug-behavior remains undefined. Evidence from the nucleus accumbens of cocaine-experienced rats suggests that decreased transporter-mediated glutamate clearance and enhanced synaptic glutamate release contribute to the potentiated glutamate signaling that underlies the enduring vulnerability to relapse. Preliminary evidence from the dorsal striatum of cocaine-experienced rats suggests that this region exhibits similar alterations to glutamate clearance and release, but it is not known whether these glutamate dynamics are associated with goal-directed or habitual control over cocaine-seeking behavior. Therefore, we trained rats to self-administer cocaine in a chained cocaine-seeking and -taking paradigm, which yielded goal-directed, intermediate, and habitual cocaine-seeking rats. We then assessed glutamate clearance and release dynamics in the DLS of these rats using two different methods: synaptic transporter current (STC) recordings of patch-clamped astrocytes and the intensity-based glutamate sensing fluorescent reporter (iGluSnFr). While we observed a decreased rate of glutamate clearance in STCs evoked with single-pulse stimulation in cocaine-experienced rats, we did not observe any cocaine-induced differences in glutamate clearance rates from STCs evoked with high frequency stimulation (HFS) or iGluSnFr responses evoked with either double-pulse stimulation or HFS. Furthermore, GLT-1 protein expression in the DLS was unchanged in cocaine-experienced rats, regardless of their mode of control over cocaine-seeking behavior. Lastly, there were no differences in metrics of glutamate release between cocaine-experienced rats and yoked-saline controls in either assay. Together, these results suggest that glutamate clearance and release dynamics in the DLS are largely unaltered by a history of cocaine self-administration on this established cocaine seeking-taking paradigm, regardless of whether the control over the cocaine seeking behavior was habitual or goal directed.

## 1. Introduction

Drug addiction is a chronic, relapsing disorder wherein individuals experience inflexible, uncontrollable drug use that persists despite adverse consequences (Vanderschuren and Everitt, 2004; Belin et al., 2009). Initially, drugs are intentionally consumed to obtain a reinforcing outcome, which reflects motivated, flexible behavior that is under goal-directed control (Vanderschuren and Everitt, 2004; Ersche et al., 2016). In contrast, as drug use continues, these drug-seeking and taking behaviors become automatically performed in response to drug-associated environmental stimuli, which is considered to reflect habitual control (Everitt and Robbins, 2005; Pierce and Vanderschuren, 2010). Over time, these habits have the ability to become compulsive, wherein drug-seeking and taking behaviors persist despite adverse consequences, which is a cardinal symptom of end-stage drug addiction (Belin et al., 2013). As such, elucidating the behavioral processes involved in the progression of drug use and their underlying neurobiological processes is a critical area of study.

Goal-directed and habitual behavior are mediated by distinct subregions within the striatum (Dolan and Dayan, 2013). Goal-directed behavior is mediated by the dorsomedial striatum (DMS) and is engaged during early behavioral learning (Yin et al., 2005a,b, 2009). In contrast, habitual behavior is mediated by the dorsolateral striatum (DLS) and is engaged after repeated behavioral practice (Packard and Knowlton, 2002; Yin et al., 2004; Yin and Knowlton, 2006; Yin et al., 2009; Dolan and Dayan, 2013). Classic studies have demonstrated that inactivation or lesion of the DMS results in early emergence of habitual actions (Yin et al., 2005a,b; Yin and Knowlton, 2006), whereas inactivation of the DLS results in maintenance of goal-directed behavior despite prolonged training (Yin et al., 2004; Yin and Knowlton, 2006). Similarly, in cocaine self-administration paradigms, transient inactivation of the DLS reverts habitual cocaine-seeking behavior back to goal-directed control (Zapata et al., 2010), attenuates cue-controlled drug-seeking (Jonkman et al., 2012), and increases sensitivity to punishment (Belin et al., 2013). To date, however, the neurobiological mechanisms within the DLS that mediate habitual drug behavior are undefined.

The development of habitual behavior is associated with enduring glutamatergic changes in the DLS (Lipton et al., 2019; Malvaez, 2020). For example, extended skill training significantly increases neuronal excitability and synaptic strength (Yin et al., 2009), suggesting that habitual behavior is mediated by potentiated glutamate signaling in the DLS. Further, disruption of postsynaptic glutamate NMDA or AMPA receptor activation during task acquisition (Palencia and Ragozzino, 2005), consolidation (Goodman et al., 2017), or performance (Corbit et al., 2014) prevents the formation or expression of habitual behavior. Thus, altered glutamate signaling mediates the development and maintenance of habitual behavior, but relatively little is known about the state of glutamate signaling in the DLS in the context of habitual drug behavior.

Astrocytes, through glutamate transporters, play a key role in regulating extracellular glutamate dynamics within the tripartite synapse (Mahmoud et al., 2019). In particular, the glutamate transporter-1 (GLT-1) is predominantly expressed on astrocytes and accounts for ~90% of glutamate clearance in the mature brain (Danbolt et al., 1992; Haugeto et al., 1996). Through the clearance of extracellular glutamate, astrocytic GLT-1 largely regulates both the time course and extracellular concentration of glutamate (Danbolt, 2001), which are critical to the modulation of neuronal excitatory signaling (Mahmoud et al., 2019). As such, the astrocytic GLT-1 system is well-poised to regulate various aspects of glutamate signaling and homeostasis that underlie addictive behavior.

To date, the majority of studies examining the effects of cocaine self-administration on glutamate signaling and clearance have focused on the nucleus accumbens (NAc), the ventral subregion of the striatum that mediates reward and motivation-based behavior (Kalivas, 2009; D’Souza, 2015). Most notably, extended cocaine self-administration and withdrawal are associated with decreased GLT-1 uptake capacity (Knackstedt et al., 2010; Trantham-Davidson et al., 2013) and decreased GLT-1 expression (Knackstedt et al., 2010; Reissner et al., 2012; Fischer-Smith et al., 2013; LaCrosse et al., 2017). Consequently, the synaptic dwell time of glutamate, synaptic spillover, and duration of glutamate signaling in the NAc are increased in response to drug cues (Niedzielska-Andres et al., 2021). In addition to altered clearance, the glutamate system is primed for enhanced synaptic release of glutamate in the NAc during cocaine reinstatement (McFarland et al., 2003; Madayag et al., 2007; Li et al., 2010). Together, these alterations in NAc glutamate clearance and release are associated with the enduring vulnerability to relapse that is central to drug addiction (Kalivas, 2009; Niedzielska-Andres et al., 2021). However, less is known about glutamate dynamics in the DLS and its role in the formation of habitual drug-seeking behavior. Interestingly, one study demonstrated increased extracellular glutamate levels in the DLS in response to acute cocaine challenge in rats withdrawn for 24 h from cocaine self-administration (Gabriele et al., 2012). In addition, GLT-1 protein expression is reportedly decreased in the DLS following extended, long-access to cocaine self-administration (Ducret et al., 2016). While these studies have begun to identify cocaine-induced alterations to the DLS glutamate system, the status of this system in the context of goal-directed verses habitual cocaine-seeking behavior remains an undefined and important area of study.

The objective of the present study, therefore, was to assess the state of the DLS glutamate system of rats characterized as being goal-directed verses habitual in their cocaine-seeking behavior, as well as in comparison to yoked-saline controls. Rats were trained in a chained cocaine self-administration paradigm that involved distinct cocaine-seeking and -taking behaviors, outcome devaluation, and an assessment of the mode of control over cocaine-seeking behavior. We then assessed glutamate clearance and release in the DLS of these rats with synaptic transporter current (STC) recordings of patch-clamped astrocytes and the intensity-based glutamate-sensing reporter (iGluSnFr). In both of these assays, we found that glutamate clearance and release were largely unchanged across goal-directed and habitual cocaine-seeking rats, as well as in comparison to yoked-saline controls. Consistent with these findings, GLT-1 protein expression in the DLS was unchanged. Overall, the present results suggest that the DLS glutamate system is largely unaltered by a history of cocaine self-administration, whether the drug-seeking behavior is under goal-directed or habitual control.

## 2. Materials and methods

### 2.1. Animals

Male Long Evans rats (300–350 g) that were surgically prepared with right jugular vein catheters were obtained from Charles River Laboratories (Wilmington, MA, United States). Catheter patency was verified upon arrival to the University of Utah animal facility and maintained by daily infusions of heparin-dextrose catheter locking solution (500 IU/50% dextrose; SAI Infusion, IL, United States). Rats also received daily prophylactic Baytril (10 mg/kg, i.v.; Norbrook, Newry, United Kingdom). Rats were single-housed in standard housing conditions. Three days before the start of cocaine self-administration training, rats were food-restricted to 25 g of standard chow per day, with *ad libitum* access to water, and were maintained on this feeding schedule throughout the experiment. Rats were fed every day following behavioral training. Rats were randomly assigned to receive either cocaine training or to serve as yoked-saline controls. Animal care, surgeries, and experimental procedures followed the *Guide for the Care and Use of Laboratory Animals* (8th Edition) and were approved by the Institutional Animal Care and Use Committee at the University of Utah. Rats used in electrophysiology experiments began self-administration training 5–7 days after arrival. Rats used in fluorescent indicator studies underwent stereotaxic surgery for infusion of iGluSnFr into the DLS 6–8 days after arrival at the University of Utah and were allowed to recover for 5–7 days before the start of cocaine self-administration training.

### 2.2. Stereotaxic surgery

Only rats used in fluorescent indicator studies underwent stereotaxic surgery for viral induction of iGluSnFr in astrocytes. Rats were anesthetized with isoflurane (2.0–2.5% induction; 1.5–2.0% maintenance) and placed in a stereotaxic apparatus (Stoelting, IL, United States). A small hole was drilled in the skull and a 28-gage infusion cannula (P1 Technologies, VA, United States) was inserted into the DLS (in mm relative to bregma: AP +0.7, ML -3.6, DV -5.0; Yin et al., 2004). A total of 3 μL of pENN.AAV1.GFAP.iGluSnFr.WPRE.SV40 (RRID:Addgene_98,930; http://n2t.net/addgene:98930; RRID:Addgene_98,930) was infused into the DLS over the course of 30 min, after which the cannula was left in place for an additional 5 min before removal to ensure maximal diffusion. Rats were given 10 mg/kg Baytril i.v. and 0.1 mL of 500 IU/mL heparin-50% dextrose catheter-Lock Solution (SAI infusions, IL, United States) on the day of surgery, as well as once daily, along with 5 mg/kg carprofen s.c., for 3 days post-surgery. For the remainder of the recovery period and throughout the cocaine self-administration training and testing, rats were given daily infusions of i.v. Baytril and the heparin-dextrose catheter-lock solution following daily training. Rats were allowed to recover from stereotaxic surgery for 3–5 days before food restriction began, and 5–7 days before self-administration training.

### 2.3. Cocaine self-administration paradigm

The cocaine self-administration paradigm, adapted from Zapata et al. (2010), was conducted in eight standard operant chambers that were enclosed in sound and light-attenuating cabinets (Coulbourn Instruments, PA, United States). One wall of the chamber had two retractable levers on the right and left sides and the opposite wall was equipped with a 3-W, 24-V house light. Graphic State 4.0 software (Coulbourn Instruments, PA, United States) was used to control chamber equipment and experimental protocols, as well as to record the number of lever presses and session time. Male Long-Evans rats were trained to press one lever (designated as the drug “taking” lever) for intravenous cocaine•HCl infusion (0.33 mg/50 μL infusion; calculated as the salt; NIDA Drug Supply Program, NC, USA) under a fixed ratio 1 (FR1) schedule. In addition to cocaine infusion, each taking-lever press was accompanied by a retraction of the taking lever, illumination of a cue light, and extinction of the house light for a 30-s time-out (TO) period. Rats were trained daily on the taking lever for 2 h or 40 infusions per session, whichever came first. After reliable taking-lever self-administration was established (2 consecutive sessions with >10 infusions/session; average intake across all rats = 9.82 mg/kg/h), rats progressed to a chained seeking-taking schedule in which an additional, different lever (designated as the “seeking” lever) was introduced. The drug-taking and -seeking levers were counterbalanced across the left and right levers in the operant chambers. The first press of the seeking lever, under a 2-s random interval (RI) schedule, resulted in retraction of the seeking lever and presentation of the taking lever. Responding on the taking lever was kept on FR1 schedule of cocaine administration, followed by the 30-s TO period. Following the TO period, the seeking lever was reinserted, and the cycle began again. This seeking-taking chained schedule is denoted as: RI(2 s)/FR1:TO(30s) or “Chain #1.” Rats underwent daily training on this schedule (3 h or 12 infusions, whichever came first) until they reached criterion of two consecutive sessions with 12 infusions/session. Rats then progressed through increasing chained schedules: Chain #2 RI(20s)/FR1:TO(120 s) and Chain #3 RI(60s)/FR1:TO(300 s) using the same training criteria. They then advanced to Chain #4 RI(120 s)/FR1:TO(600 s), which continued for six sessions (12 infusions/session), at which point all rats had achieved stable responding (<20% variability in drug infusions; average intake per Chain 4 session across all rats = 4.6 mg/kg/h). Yoked-saline controls received response-independent saline infusions identical to that of their paired experimental rat and lever-pressing had no scheduled consequences.

Once stable chained responding was established, rats progressed to 13 days of outcome devaluation; that is, the taking lever was available, but no cocaine was delivered upon lever-pressing, thereby devaluing the drug-taking lever via outcome omission. After the last day of outcome devaluation, seeking behavior was assessed under these “devalued” conditions in a 5-min test session. In this seeking test, only the seeking-lever was available and responding did not result in any scheduled consequences. Following the devalued seeking test, the taking-lever was then re-valued across two daily sessions identical to the initial FR1 taking lever training sessions (2 h or 40 infusions, whichever came first). The following day, another 5-min seeking test was conducted, but now under “valued” conditions. Comparing the number of cocaine-seeking lever presses made under the “devalued” vs. the “valued” test conditions gives insight into the sensitivity of drug-seeking to outcome devaluation, which enables the classification of cocaine-seeking behavior as either under goal-directed or habitual control (Zapata et al., 2010; Lerner, 2020). A “cocaine-seeking score” was determined for each rat as the number of seeking responses made under the *devalued* test condition expressed as a percentage of seeking responses made under the *valued* test condition. Rats with cocaine-seeking scores ≤70% were classified as having “goal-directed” cocaine-seeking behavior and scores ≥80% were classified as “habitual,” similar to that previously reported by Zapata et al. (2010). As a small percentage of rats had cocaine-seeking scores that fell between 71–79%, we termed these rats as “intermediate” and analyzed them as a separate group. All rats were sacrificed within 1–4 days following completion of the final seeking test, given the throughput of the electrophysiological recordings and iGluSnFr imaging. We limited this time course to 1–4 days after the testing as the seminal work on incubation of cocaine craving showed no significant changes in drug seeking over days 1–4 (Grimm et al., 2001) and subsequent studies of incubation variably use 1–4 days as “early” withdrawal time points. The 1–4 day period should therefore mitigate potential confounding effects of withdrawal and incubation of craving on the dependent measures.

### 2.4. Brain slice preparation

From the point of brain slice preparation onward through final data analysis, the experimenter was blinded to the treatment group (saline or cocaine) and behavioral classification (goal-directed, intermediate, habitual) of the rats. Within 4 days after the final seeking test, rats were anesthetized with 3% isoflurane or sodium pentobarbital (50 mg/kg) and immediately decapitated. The brain was then divided into two hemispheres longitudinally. For electrophysiology recordings, sagittal brain slices (400 μm) containing the DLS were collected in an oxygenated ice-cold NMDG-HEPES cutting solution (in mM: 92 NMDG, 2.5 KCl, 1.2 NaH_2_PO_4_, 30 NaHCO_3_, 20 HEPES, 25 glucose, 2 thiourea, 5 Na-ascorbate, 3 Na-pyruvate, 10 MgSO_4_, and 0.5 CaCl_2_). Slices were then transferred to a pre-warmed (32–24°C) holding chamber containing NMDG-HEPES for 30 min as a protective recovery period. Stepwise, a Na^+^ spike-in procedure was carried out according to an optimal age-dependent schedule (Ting et al., 2014). After 30 min, slices were transferred to a holding chamber containing room-temperature oxygenated HEPES-aCSF holding solution (in mM: 92 NaCl, 2.5 KCl, 1.2 NaH_2_PO_4_, 30 NaHCO_3_, 20 HEPES, 25 glucose, 2 thiourea, 5 Na-ascorbate, 3Na-pyruvate, 2 MgSO_4_, and 2 CaCl_2_). Slices were then transferred to a pre-warmed (30°C) holding chamber for 20 min prior to recording for astrocyte labeling with 0.5 μM SR101 (Sigma-Aldrich, MO, United States) in recording aCSF (in mM: 119 NaCl, 2.5 KCl, 1.25 NaH_2_PO_4_, 24 NaHCO_3_, 12.5 glucose, 2 MgCl_2_, and 2 CaCl_2_) (Takahashi et al., 2010). For iGluSnFr imaging, coronal brain slices (400 μm) containing the right hemisphere DLS were cut on a Vibratome 3,000 (Vibratome, MO, United States) in an ice-cold, sucrose solution (in mM: 185 sucrose, 2.5 KCl, 1.2 NaH_2_PO_4_, 25 NaHCO_3_, 25 glucose, 10 MgSO_4_, 0.5 CaCl_2_). Brain slices were then transferred to a recovery chamber containing oxygenated aCSF at room temperature (in mM: 126 NaCl, 2.5 KCl, 1 NaH_2_PO_4_, 26 NaHCO_3_, 10.5 glucose, 1.3 MgSO_4_, 2 CaCl_2_). All slices, regardless of slice method, were allowed to recover for a minimum of 1 h before recording or imaging. All solutions were bubbled with 95% O_2_/5% CO_2_ throughout the experiment, pH corrected to 7.30–7.35, and adjusted for an osmolarity between 295–300 mOsm. The non-injected hemisphere from iGluSnFr-transfected rats was flash-frozen in 2-methylbutane (MilliporeSigma, MA, United States) and stored at −80°C for later use in western blot analyses.

### 2.5. Electrophysiology

Whole-cell patch-clamp recordings of astrocytes were obtained by recording in the voltage-clamp configuration through a Multiclamp 700B amplifier, a Digidata 1440A data acquisition board, and pClamp10 software (Molecular Devices, CA, USA). Slices were visualized with a 40x water immersion objective (NA 0.8; Carl Zeiss, Thornwood, NY) using infrared differential interference contrast (IR-DIC) microscopy on an upright Axioskop2 microscope (Carl Zeiss, Thornwood, NY) prior to specific astrocyte identification. Only cells displaying SR101 fluorescence (excitation 586 nm, emission 605 nm) deep in the tissue were targeted for electrophysiology with pipettes containing a K^+^ gluconate intracellular solution (in mM: 120 K gluconate, 20 HEPES, 10 EGTA, and 0.2 Na_2_GTP), pH corrected to 7.28–7.33, and osmolarity between 290–295 mOsm. Astrocytes were voltage-clamped at -70 mV and further distinguished from neurons by their hyperpolarized resting membrane potential, low input resistance, and lack of voltage-dependent inward currents at depolarized potentials. Resting membrane potential was periodically determined in the *I *= 0 mode and monitored throughout recordings. Cells with variations in resting membrane potential greater than ±10 mV were excluded from analyses. Membrane and access resistance were monitored and only stable recordings were included in the study. Upon successfully obtaining the whole-cell patch configuration, a pharmacological cocktail containing BaCl_2_ (200 μM), APV (50 μM), CNQX (10 μM), and picrotoxin (50 μM) was perfused for 15 min to isolate STCs.

A bipolar nichrome/formvar stimulating electrode placed 100–200 μm from the patch pipette in the DLS was used to deliver a single pulse or a high frequency stimulation train (10 pulses, 100 Hz). Stimulation strength was set as the intensity at which stimulation produced a half-maximal response amplitude following an input–output curve of increasing stimulation intensities, and this strength was used for all subsequent STC recordings. A maximum of two astrocytes were patched per slice. Signals were acquired at 10 kHz and filtered at 2 kHz. All response amplitude and time course analyses were performed on averaged, baseline corrected STCs in ClampFit (Molecular Devices, CA, United States). Rise times were calculated as the time it took for the current to rise from baseline to peak amplitude. Decay tau values were obtained by curve fitting the decay phase to a second order standard exponential equation, with only fast decay tau values included for analyses. Curves were fit from just after peak STC amplitude of the single pulse or the final (10th) pulse of HFS, to the end of the trace when currents have returned to baseline. Half-width was calculated as the time between the 50% values of the rise and decay times. Pulse ratios were determined by STC amplitude of the 10^th^ pulse to the 1st pulse of the HFS train. All analyses, with the exception of peak amplitude and pulse ratios, were also performed in traces normalized to peak current.

### 2.6. iGluSnFr imaging

iGluSnFr fluorescence was excited by a 470-nm LED light (Excelitas, Mississauga, Canada) and captured with an Axiocam 702 monochrome camera coupled with ZEN 3.1 (blue edition) software (Zeiss, MA, United States). All video recordings of evoked iGluSnFr signals were kept at constant imaging settings within the Zen software to allow for an image acquisition rate of 92 Hz (ROI size: 480 × 510; Exposure time: 10 ms; Intensity: 100%; Binning: 5 × 5; Gain: 2x). A bipolar, nichrome/formvar electrode was positioned within the DLS at a depth of ~100 μm below the tissue surface to deliver a 1-mA double-pulse stimulation (two, 0.1 ms pulses, 10 ms apart). Three to five stimulation trials, conducted in the same location and spaced 1 min apart, were averaged together to produce one “evoked iGluSnFr response” that was used for analysis. After the final double-pulse stimulation trial was conducted in a given area, the electrode was kept in the same position and three stimulation trials were conducted with 1 mA high frequency stimulation (HFS; 0.1 ms pulses delivered at 10 Hz for 1 s) following the same trial protocol as above. Each stimulation trial was video recorded for 15 s, and included 4 s of baseline iGluSnFr fluorescence. Rats with low levels of iGluSnFr expression, no expression, or expression outside the DLS were not included in the final data analysis.

Evoked iGluSnFr responses were analyzed in ImageJ by placing a 158×158 pixel ROI (30×30 unit in ImageJ) at the location surrounding the highest peak iGluSnFr signal, typically adjacent to the stimulating electrode. Fluorescence intensity changes were baseline-corrected and used for analysis of decay kinetics in GraphPad Prism (GraphPad Software Inc., CA, United States). Decay tau was calculated by fitting the evoked iGluSnFr trace from the peak dF/*F* value to one-second post-peak with a one-phase, non-linear regression (Parsons et al., 2016; Pinky et al., 2018). For HFS trials, the response was fit from the final peak dF/F value in the HFS train to one-second post-peak (Parsons et al., 2016). Area under the curve (AUC) was calculated from the peak dF/F or final peak dF/F to one-second post-peak for both double-pulse stimulation and HFS, respectively.

A series of iGluSnFr control experiments were conducted in rats that were not enrolled in the chained cocaine self-administration paradigm, but were imaged within the same timeframe that experimental rats were imaged (approximately 6 weeks post-stereotaxic surgery). For temperature control experiments, evoked iGluSnFr responses were recorded in the same location within the DLS at 24°C and 32°C. Three stimulation trials (1 mA double-pulse stimulation) were first recorded at 24°C, and then three additional stimulation trials were recorded in the same location at 32°C. Bath temperature within the recording chamber was monitored and maintained with an in-line heater system (TC-324C, Warner Instruments, MA, United States). For all pharmacological control experiments, three baseline iGluSnFr responses (1 mA double-pulse) were first evoked in the DLS; next, three iGluSnFr responses (1 mA double-pulse) were evoked in the same location within the DLS in the presence of the pharmacological agent after the specified waiting period. For tetrodotoxin (TTX) experiments, 1 μM TTX citrate (Abcam, Cambridge, United Kingdom) in aCSF was perfused into the recording chamber for 15 min before imaging evoked iGluSnFr responses under TTX conditions. For TBOA experiments, the non-selective glutamate transporter inhibitor DL-threo-β-Benzyloxyaspartic acid (DL-TBOA, 100 μM) (Tocris, MN, United States) was perfused into the recording chamber for 10 min before imaging evoked iGluSnFr responses under TBOA conditions. For all experiments, three stimulation trials (1 mA double-pulse), spaced 1-min apart, were conducted for each experimental condition and were averaged together for later analysis.

### 2.7. Western blot

Non-injected hemispheres from iGluSnFr-transfected rats were used for western blot analysis of GLT-1 expression in the DLS. The frozen hemisphere was sectioned to bregma 1.44 mm at −16°C in a cryostat (Leica). Punches of the DLS (~1-mm^3^) were taken and then sonicated in 2% SDS with protease inhibitor (cOmplete™ Mini, Sigma Roche; #11836153001), and centrifuged for 10 min (10,000 g at 4°C). The supernatant was collected and used for western blotting. Equal amounts of total protein, as determined by BCA assay, were prepared in 4x Laemmli sample buffer (Bio-Rad, #1610747). Samples were heated at 70°C for 10 min and 15 μL loaded onto 4–15% TGX gels (Bio-Rad Criterion #5671085). One well contained the ladder for determining molecular weights (Bio-Rad Precision Plus Protein Standard). Gels were run at 75 V for 15 min and then 125 V for 45–70 min. Gels were then transferred to nitrocellulose membranes at 100 V for 1.5 h at 4°C. Blots were stained with 1x Ponceau stain for 2 min and imaged on the FluorChem M (Protein Simple, Bio-Techne) to determine total protein in each lane. Gels were then blocked for 1 h in blocking solution (TBS-T) and incubated with anti-GLT1 primary antibody overnight at 4°C (1:2000, Millipore #AB1783, RRID: AB_90949). After washing in TBS-T, blots were incubated with Goat anti-guinea pig HRP secondary antibody (1:10,000; Jackson ImmunoResearch Laboratories #106–035–003, RRID: AB_2337402) for 1 h at room temperature. Blots were developed using the enhanced chemiluminescence reagent Western Lighting Plus Luminol Kit (Perkin Elmer NEL103001) and scanned using FluorChem M. The fluorescent intensity of each band was normalized to Ponceau as a loading control, and the resulting ratio was used to assess protein concentrations.

### 2.8. Statistical analysis

All statistical analyses were performed in GraphPad Prism (GraphPad Software Inc., CA, United States). For all analyses, alpha was set to 0.05. D-Agostino & Pearson normality tests were performed on all data sets to determine if the data was normally distributed. If the data were normally distributed (*p* > 0.05), parametric tests were used, including: one-way ANOVA, paired t-tests, unpaired t-tests, and Pearson correlation. If the data were not normally distributed (*p* < 0.05), nonparametric versions of the aforementioned tests were used, including: Kruskall Wallis test, Mann–Whitney *U* test, Wilcoxon matched-pairs signed rank test, and Spearman correlation, respectively. As noted above, all electrophysiology recordings, iGluSnFr imaging, and data analyses were conducted with the experimenter blinded to the treatment and behavioral classification of the subject.

## 3. Results

### 3.1. Cocaine-seeking and -taking behavior

**Figure 1:**
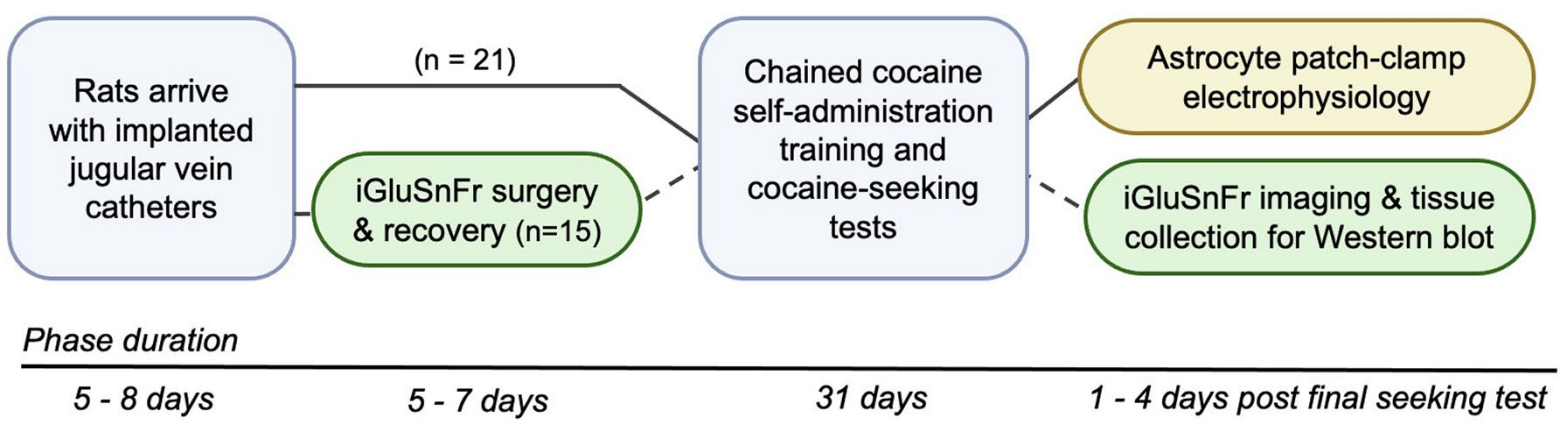
Schematic of experimental timeline. All rats arrived with implanted jugular vein catheters and were given 5–8 days to acclimate to animal facility housing. Rats in the electrophysiology cohort (*n* = 21, black lines) were directly advanced into the chained cocaine self-administration paradigm, whereas rats in the imaging cohort (*n* = 15, dashed lines) first underwent stereotaxic surgery to transfect DLS astrocytes with iGluSnFr and then were advanced to the self-administration paradigm 5–7 days later. Upon completion of the cocaine self-administration paradigm and testing for cocaine seeking under devalued and valued conditions, electrophysiological recordings, iGluSnFr imaging, and tissue collection were conducted within 1–4 days of the final cocaine-seeking test.

**Figure 2:**
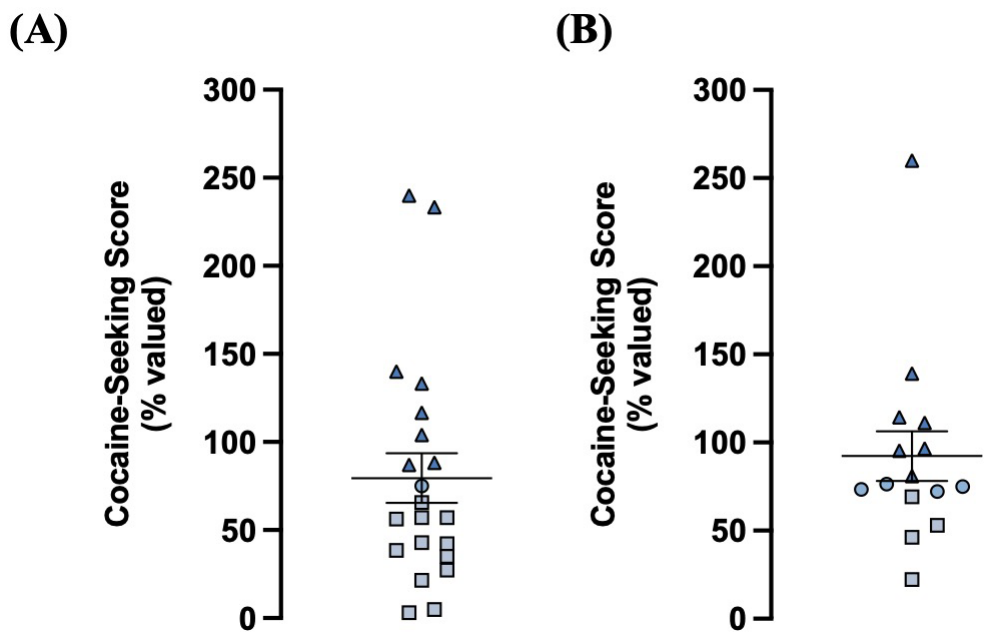
A chained cocaine seeking-taking self-administration paradigm reliably establishes rats with goal-directed and habitual control over cocaine-seeking behavior. **(A)** Distribution of cocaine-seeking scores from non-transfected animals (*n* = 21; goal-directed: *n* = 12, intermediate *n* = 1, habitual: *n* = 8). **(B)** Distribution of cocaine-seeking scores from iGluSnFr-transfected animals (*n* = 15; goal-directed: *n* = 4, intermediate: *n* = 4, habitual: *n* = 7). Cocaine-seeking scores (devalued seeking responses expressed as a percentage of valued seeking responses) are classified as goal- directed (≤70%, squares), intermediate (71–79%, circles), and habitual (≥80%, triangles). Data expressed as the mean ± SEM.

Rats in the electrophysiology (*n* = 21) and iGluSnFr imaging (*n* = 15) cohorts were enrolled in cocaine self-administration training and DLS glutamate dynamics were assessed within 1–4 days after the final cocaine-seeking test (Figure 1). We used a chained cocaine self-administration paradigm, adapted from Zapata et al. (2010), to train distinct cocaine-seeking and -taking behaviors. Importantly, the number of seeking-lever responses made under devalued and valued conditions were used to calculate the cocaine-seeking score, which classified rats as either goal-directed (≤ 70%) or habitual (≥ 80%) in their cocaine-seeking behavior (Figure 2). We observed a subset of iGluSnFr-transfected rats (*n* = 4) whose cocaine-seeking scores fell between 71–79%, which we termed “intermediate.” As the non-transfected group only had one intermediate cocaine-seeking rat, it was excluded from subsequent STC analyses.

Both non-transfected and iGluSnFr-transfected rats readily met criteria for all cocaine-seeking and taking responses throughout training, extinguished cocaine-taking upon devaluation, and reinstated their cocaine-taking behavior upon revaluation (Supplementary Figures S1 and S2). Moreover, there was no significant difference in the average cocaine-seeking score between iGluSnFr-transfected and non-transfected rats (*Mann–Whitney U = 117.5, p = 0.21*; Supplementary Figure S1A). Next, there was no main effect of transfection on the number of taking-lever presses made across devaluation sessions (*two-way RM ANOVA, F_(1,34)_ = 0.0003, p = 0.99*; Supplementary Figure S1B). There was, however, a significant transfection x day interaction for the number of seeking-lever presses made during chained training (*two-way RM ANOVA, F_(11,418)_ = 3.49, p = 0.0001*; Supplementary Figure S1C), with iGluSnFr-transfected rats making significantly more seeking-responses than non-transfected rats during the first 5 sessions, but not the final session, of chain 4 training (*Sidak’s multiple comparisons test, sessions 1–5: p < 0.01, session 6: p =  0.07*). However, there was no significant correlation between the average number of chain 4 seeking-responses and cocaine-seeking score for both non-transfected rats (*Spearman r = −0.30, p = 0.19*; Supplementary Figure S1D) and iGluSnFr-transfected rats (*Spearman r = 0.13, p = 0.65*; Supplementary Figure S1E). Similarly, iGluSnFr-transfected rats had a significantly higher average number of taking-lever presses than non-transfected rats during both the initial FR1 (*Mann–Whitney U = 47, p < 0.001*; Supplementary Figure S2A) and revaluation FR1 (*Mann–Whitney U = 39.5, p < 0.0001*; Supplementary Figure S2B) training sessions. However, the average number of taking-lever presses made in either stage did not correlate with cocaine-seeking score for both non-transfected rats (Initial: *Spearman r = −0.42, p = 0.052*; Revaluation: *Spearman r = −0.26, p = 0.25*; Supplementary Figure S2C) and iGluSnFr-transfected rats (Initial: *Spearman r = 0.06, p = 0.84*; Revaluation: *Spearman r = −0.12, p = 0.66*; Supplementary Figure S2D). In addition, both non-transfected and iGluSnFr-transfected rats reinstated taking-lever presses to at or above the level of their initial FR1 training (Supplementary Figure S2E). Taken together, while we observed some training differences between transfected and non-transfected rats during cocaine self-administration sessions, these differences did not impact the generation or classification of goal-directed, intermediate, and habitual cocaine-seeking behavior in either group. Moreover, as we reliably generated goal-directed and habitual cocaine-seeking rats in both groups, we proceeded to assess the state of the DLS glutamate system of these rats with their respective assays of glutamate clearance and release.

### 3.2. Analyses of synaptic transporter currents in astrocytes reveal minimal changes in glutamate clearance in cocaine-experienced rats

**Figure 3:**
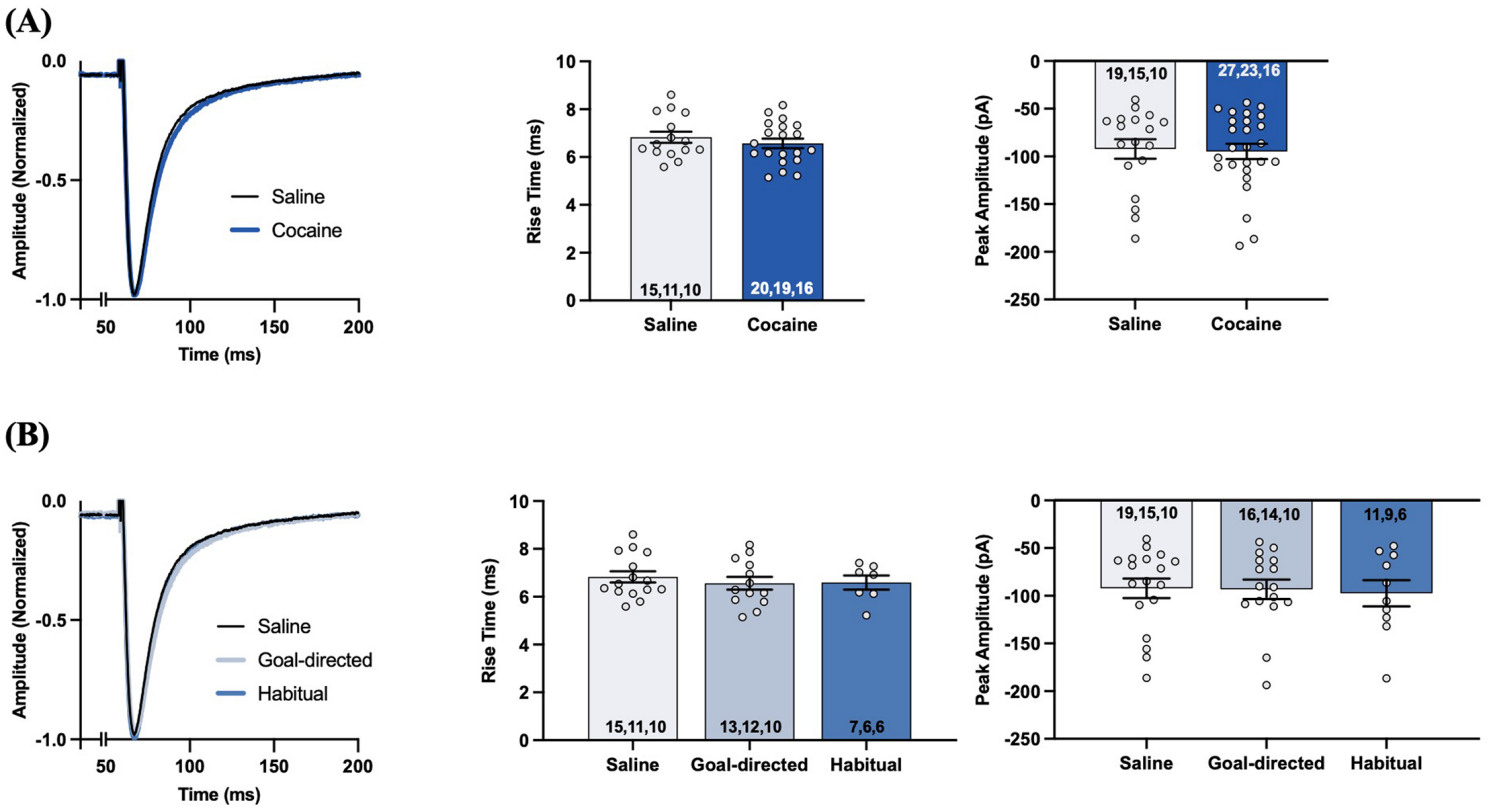
Synaptic transporter currents (STCs) in the DLS of acute brain slices from cocaine-experienced animals display no differences in the rise time or the peak amplitude of the STCs in astrocytes. **(A)** Representative peak-normalized STCs from slices of cocaine-experienced rats and yoked-saline controls following single-pulse stimulation at an intensity that produced a half maximal response amplitude. The STC rise time (0–100%) is quantified in the associated bar graph, along with peak STC amplitude (unpaired *t*- test, *p* > 0.05). **(B)** Representative peak-normalized STCs from slices of saline, goal-directed, and habitual rats following single-pulse stimulation, with the corresponding quantified STC rise time and peak amplitude (one-way ANOVA with Dunnet’s *post hoc* test, Kruskal-Wallis test, *p* > 0.05, respectively). Data are expressed as the mean ± SEM. Sample sizes for each group are denoted in each corresponding bar as: number of cells, number of slices, number of animals.

We recorded evoked STCs of voltage-clamped astrocytes in the DLS of acute brain slices obtained from yoked-saline controls and rats classified as goal-directed or habitual in their cocaine-seeking to assess astrocyte-mediated glutamate clearance. Each aspect of evoked STCs reflects various components of glutamate signaling as assessed through glutamate transporters on the astrocytes (Figure 3). The rise phase of the STC reflects the concerted uptake activity of glutamate transporters expressed on the astrocyte membrane in response to glutamate, whereas STC amplitude reflects the amount of glutamate released at the synapse and sensed by the astrocyte in question (Diamond and Jahr, 2000; Diamond, 2005; Tzingounis and Wadiche, 2007). STC rise times were not significantly different between slices obtained from yoked control (6.8 ± 0.2 ms; *n* = 19 cells, 15 slices, 10 rats) or cocaine-experienced (6.6 ± 0.2 ms; *n* = 27 cells, 23 slices, 16 rats) rats (*unpaired t-test, t = 0.85, p = 0.40;* Figure 3A). STC peak amplitudes were also not significantly different between slices from yoked-saline (−92.2 ± 10.2pA) and cocaine-experienced (−94.8 ± 8.0pA) rats when stimulated at an intensity that produced a half-maximal response amplitude (*unpaired t-test, t = 0.20, p = 0.84;* Figure 3A). Similarly, there were no significant differences in STC rise times recorded in slices from goal-directed (6.6 ± 0.3 ms; *n* = 16 cells, 14 slices, 10 rats) vs. habitual (6.6 ± 0.3 ms; *n* = 11 cells, 9 slices, 6 rats) classifications and in comparison to yoked-saline controls (*Kruskall-Wallis statistic = 0.30, p = 0.86;* Figure 3B). Peak amplitudes were also similar between slices from goal-directed (−93.2 ± 10.2pA) vs. habitual (−97.4 ± 13.8pA) cocaine-seeking rats, as well as yoked-saline controls (*one-way ANOVA: F_(2, 41)_ = 0.05, p = 0.95;* Figure 3B). Therefore, STC rise times and amplitudes from astrocytes in the DLS of acute brain slices suggest that there are no disruptions in transporter-mediated glutamate uptake into astrocytes or the amount of glutamate released at the synapse in cocaine-experienced rats regardless of their behavioral classification.

**Figure 4:**
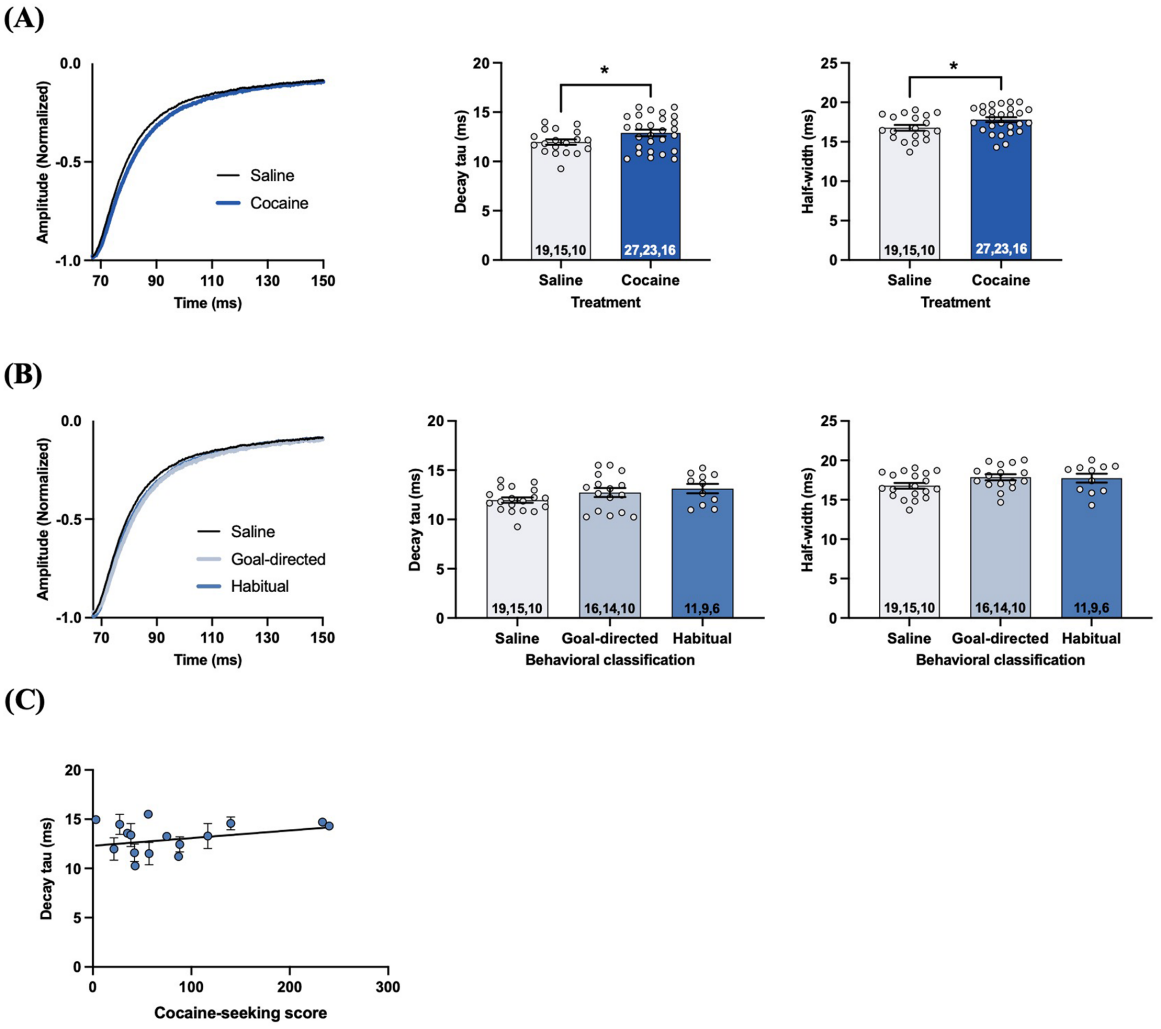
STCs in the DLS of slices obtained from cocaine-experienced animals display slower glutamate clearance kinetics. **(A)** Representative peak-normalized STC response decay phase, decay tau rate constant, and STC half-width following single pulse stimulation in the DLS of acute brain slices from yoked-saline and cocaine-experienced animals (unpaired t-test, *p* < 0.05). **(B)** Representative peak-normalized STC response decay phase, decay tau, and STC half-width in slices from yoked-saline rats or rats classified as goal-directed and habitual in their cocaine seeking (one-way ANOVA, with Dunnet’s *post hoc* test, *p* > 0.05). **(C)** Relationship between decay tau and cocaine-seeking score (Spearman correlation, *p* > 0.05). Data fit with a linear regression line of best fit. Bar graphs expressed as the mean ± SEM. ^*^p < 0.05. Sample sizes for each group **(A,B)** are denoted in each corresponding bar as: number of cells, number of slices, number of animals.

The time course of STCs reflects the rate of glutamate clearance from the synaptic cleft, with the decay phase being proportional to the decrease in the amount of extracellular glutamate available for transport (Diamond and Jahr, 2000; Diamond, 2005; Tzingounis and Wadiche, 2007). Analysis of the decay tau and half-width values of STCs in acute brain slices taken from cocaine-experienced and yoked-saline control rats revealed significantly slower decay kinetics in DLS astrocytes of rats with a history of cocaine self-administration. Specifically, decay tau values from cocaine-experienced rats (12.9 ± 0.3 ms) were significantly slower than those in yoked-saline controls (11.9 ± 0.3 ms; *unpaired t-test, t = 2.03, p < 0.05;* Figure 4A). Half-width values were also significantly longer in cocaine-experienced rats (17.8 ± 0.3 ms) in comparison to yoked-saline controls (16.7 ± 0.3 ms; *unpaired t-test, t = 2.15, p < 0.05;* Figure 4A). Together, STC decay kinetics indicate a significantly slower rate of glutamate clearance in astrocytes of cocaine-experienced rats. However, when STC decay kinetics were assessed in accordance with behavioral classifications of cocaine-seeking rats, there were no overall differences between the yoked-saline, goal-directed, and habitual cocaine-seeking groups (Figure 4B). Specifically, STC decay tau values from goal-directed (12.7 ± 0.5 ms) or habitual (13.1 ± 0.5 ms) classification groups did not significantly differ from each other or from yoked-saline controls (11.9 ± 0.3 ms; *one-way ANOVA: F_(2, 43)_ = 2.26, p = 0.12;* Figure 4B). STC half-width values were also not significantly different between slices from rats classified as goal-directed (17.8 ± 0.4 ms) or habitual (17.7 ± 0.5 ms) or from yoked-saline controls (16.7 ± 0.3 ms; *one-way ANOVA: F_(2, 43)_ = 2.28, p = 0.11;* Figure 4B). Moreover, there was no significant correlation between decay tau values and cocaine-seeking score (*Spearman r = 0.035, p = 0.90;* Figure 4C). Together, these data suggest that rats with a history of cocaine self-administration, regardless of whether their cocaine-seeking behavior is under goal-directed or habitual control, have a statistically slower rate of glutamate clearance in the DLS.

**Figure 5:**
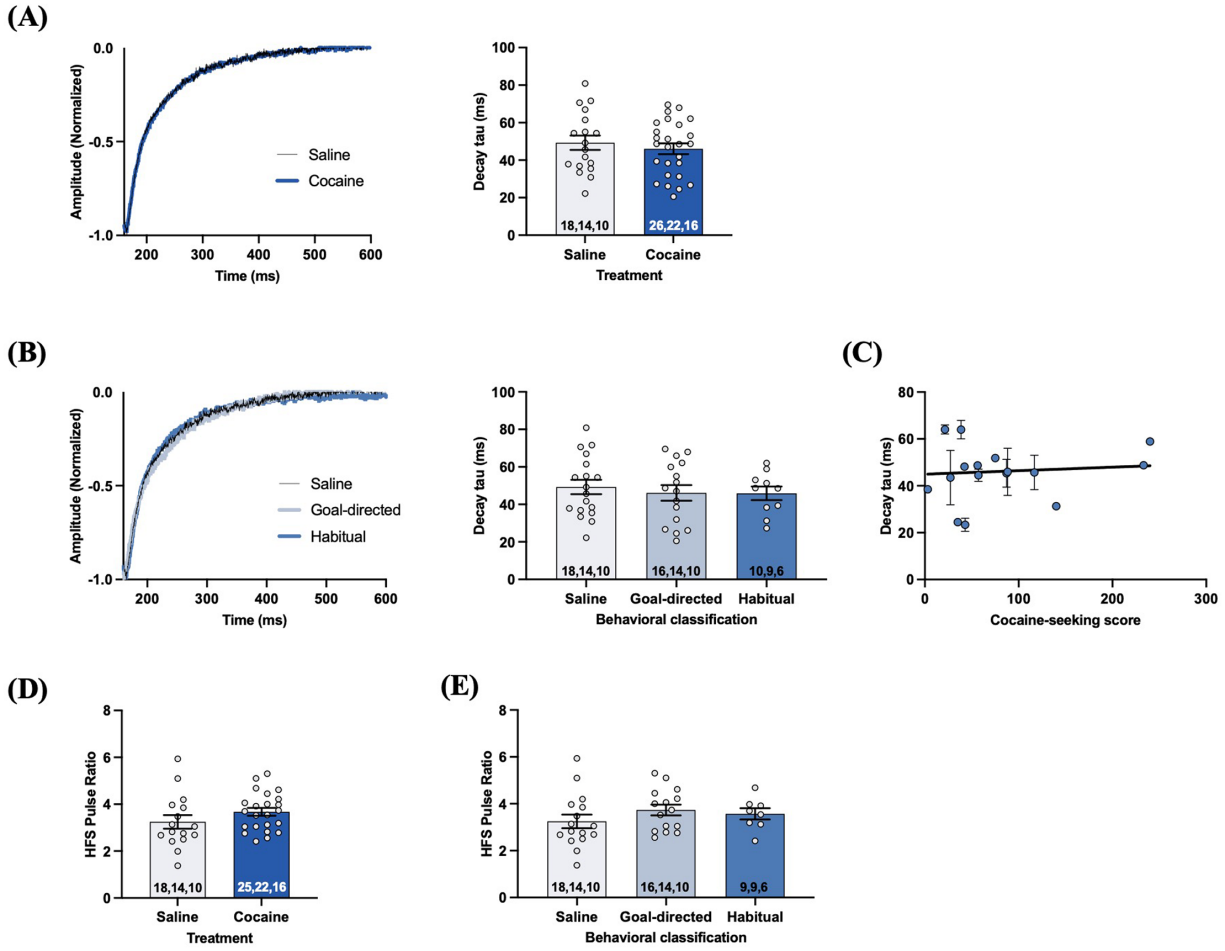
STCs following HFS in slices obtained from cocaine-experienced animals display no differences in glutamate clearance or presynaptic release in the DLS. **(A)** Representative STC decay phase, peak-normalized to the 10th stimulus, following HFS (10 pulses, 100 Hz) in slices from yoked-saline control and cocaine-experience animals. HFS STC decay tau from slices of saline and cocaine- treated animals (unpaired *t*-test, *p* > 0.05). **(B)** Representative STC decay phase following HFS in slices from yoked-saline controls and rats classified as goal-directed or habitual in their cocaine-seeking behavior and corresponding decay tau (one-way ANOVA with Dunnet’s *post hoc* test, *p* > 0.05). **(C)** Relationship between HFS decay tau and cocaine-seeking score (Spearman correlation, *p* > 0.05). Data fit with a linear regression line of best fit. **(D)** HFS pulse ratio (10th: 1st pulse amplitude) between slices from yoked-saline and cocaine self- administering animals (unpaired *t*-test, *p* > 0.05). **(E)** HFS pulse ratio between slices from yoked-saline controls and rats classified as goal-directed and habitual in their cocaine-seeking behavior (one-way ANOVA with Dunnet’s *post hoc* test, *p* > 0.05). Bar graphs expressed as the mean ± SEM. Sample sizes for each group **(A,B,D,E)** are denoted in each corresponding bar as: number of cells, number of slices, number of animals.

The difference in decay tau values of astrocyte STCs evoked by single-pulse stimulation in slices from cocaine-experienced and yoked-saline rats may not fully reflect the extent of possible biologically meaningful changes. Indeed, STCs resulting from different physiologically relevant synaptic events may be differentially affected. For example, a high frequency stimulation (HFS) protocol (10 pulses, 100 Hz) which elicits large amounts of synaptically-released glutamate has previously been shown to reveal impairments in glutamate clearance that were not apparent, or well-defined, under low frequency conditions (*c.f.*, Diamond and Jahr, 2000; Tanaka et al., 2013; Umpierre et al., 2019). Therefore, we also evoked STCs using the HFS stimulation protocol. Unexpectedly, decay tau values from STCs evoked with the HFS protocol did not significantly differ between slices obtained from yoked-saline (49.2 ± 3.8 ms) and cocaine-experienced rats (46.1 ± 2.9 ms; *unpaired t-test, t = 0.69, p = 0.*49; Figure 5A). Likewise, decay tau values of STCs from rats classified as goal-directed (46.2 ± 4.2 ms) or habitual (45.9 ± 3.6 ms) in their cocaine-seeking behavior were not significantly different relative to each other or to yoked-saline controls (49.2 ± 3.8 ms; *one-way ANOVA: F_(2, 41)_ = 0.23, p = 0.79*; Figure 5B). Also, there was no significant correlation between HFS decay tau values and cocaine-seeking scores (*Spearman r = 0.15, p = 0.57;* Figure 5C). Together, these data suggest that DLS glutamate clearance following HFS is unaffected by cocaine self-administration, as well as the nature of control over the cocaine-seeking behavior. Finally, we also assessed short term plasticity mechanisms of presynaptic facilitation by analyzing the STC pulse amplitude ratio for the 10^th^: 1^st^ pulse of the HFS train (Umpierre et al., 2019). HFS pulse ratios were not significantly different between yoked-saline (3.3 ± 0.3) and cocaine-experienced rats (3.6 ± 0.2; *unpaired t-test, t = 1.37, p > 0.05*; Figure 5D). Likewise, further sub-group analysis of HFS pulse ratios from rats classified as goal-directed (3.7 ± 0.2) or habitual (3.6 ± 0.2) in their cocaine-seeking behavior did not reveal any significant differences relative to each other or to the yoked-saline controls (3.3 ± 0.3; *one-way ANOVA: F_(2, 36)_ = 0.99, p = 0.*38; Figure 5E). These findings suggest that glutamate release and presynaptic facilitation in the DLS is also unaltered in cocaine-experienced rats, regardless of apparent goal-directed or habitual control over their cocaine-seeking behavior.

### 3.3. Evoked iGluSnFr response kinetics in the DLS are unaltered in cocaine-experienced rats

Next, we investigated extracellular glutamate dynamics using the intensity-based glutamate-sensing fluorescent reporter (iGluSnFr) (Marvin et al., 2013). Unlike STCs, iGluSnFr enables the measurement of rapid glutamate release and clearance dynamics at the regional level and can be expressed in a cell-type specific manner (Marvin et al., 2013). As the fine processes of astrocytes are in close contact with the pre- and post-synaptic components of excitatory synapses (Mahmoud et al., 2019), we selectively expressed iGluSnFr under the GFAP promotor in the DLS and confirmed selective expression by astrocytes based on the morphology of the cells expressing the iGluSnFr (Supplementary Figure S3A). The kinetics of evoked iGluSnFr responses reflect different properties of extracellular glutamate dynamics (Supplementary Figures S3B,C), including the rate of glutamate clearance (decay tau) and the magnitude of evoked synaptic glutamate release (peak signal), and the total amount of extracellular glutamate sensed by iGluSnFr-expressing cells (area under the curve; AUC) (Marvin et al., 2013, Parsons et al., 2016, Koch et al., 2018; Pinky et al., 2018; Parker et al., 2021). Control experiments using bath-applied tetrodotoxin (TTX) abolished evoked iGluSnFr responses, confirming that evoked iGluSnFr signals occur in response to local stimulation-induced, synaptic glutamate release (Supplementary Figure S3D). Next, bath-application of the non-specific glutamate transporter antagonist DL-TBOA decreased the rate of decay of the evoked-iGluSnFr response (Supplementary Figure S3E), demonstrating that iGluSnFr imaging is capable of detecting changes in glutamate clearance resulting from altered glutamate transporter function. Finally, the decay rate of evoked iGluSnFr responses was faster when the bath temperature was increased to 32°C, as compared to evoked responses measured at 24°C (Supplementary Figure S3F). Importantly, each of these iGluSnFr control experiments have been previously performed to verify iGluSnFr functioning (Parsons et al., 2016). Together, these control experiments demonstrate that iGluSnFr is properly functioning in our experimental preparation and is capable of detecting both increases and decreases in glutamate clearance as well as changes in clearance arising from altered glutamate transporter function.

**Figure 6:**
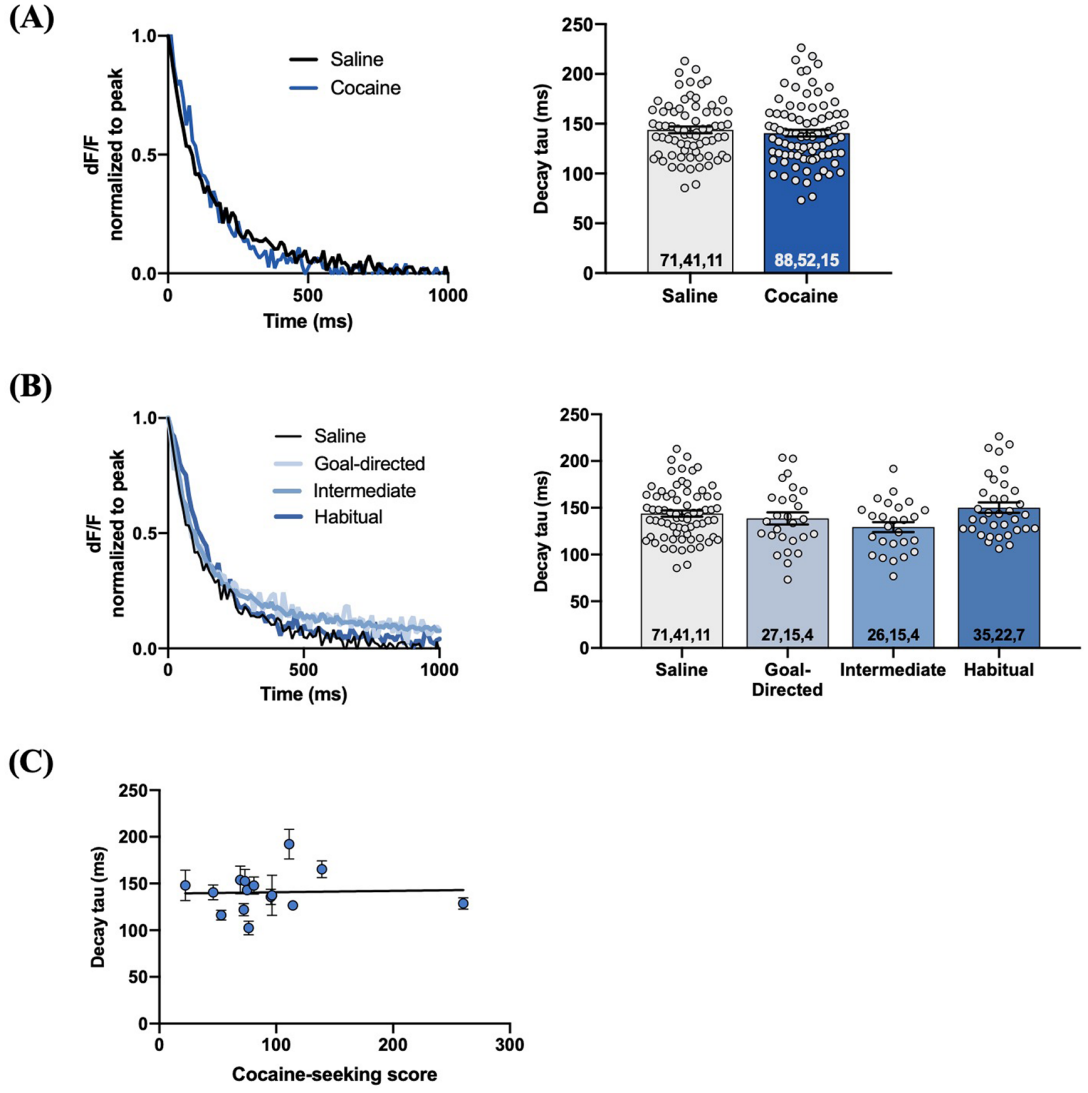
Evoked iGluSnFr responses in the DLS of slices from cocaine-experienced animals display no differences in glutamate clearance rate. **(A)** Representative peak-normalized iGluSnFr response decay following 1 mA double pulse stimulation from slices of yoked-saline and cocaine-experienced animals. Decay tau quantified in the associated bar graph (unpaired t-test, *p*>0.05). **(B)** Representative iGluSnFr response decay and decay tau values of yoked-salibe controls and goal-directed, intermediate, and habitual classifications (one-way ANOVA, *p*=0.05). **(C)** Relationship between cocaine-seeking score and decay tau of evoked iGluSnFr responses (Spearman correlation, *p* > 0.05); data fit with a linear regression line of best fit. iGluSnFr responses were evoked with 1 mA double-pulse stimulation. Bar graphs expressed as mean ± SEM. Sample sizes for each group **(A,B)** are denoted in each corresponding bar as: number of averaged responses, number of slices, number of animals.

**Figure 7:**
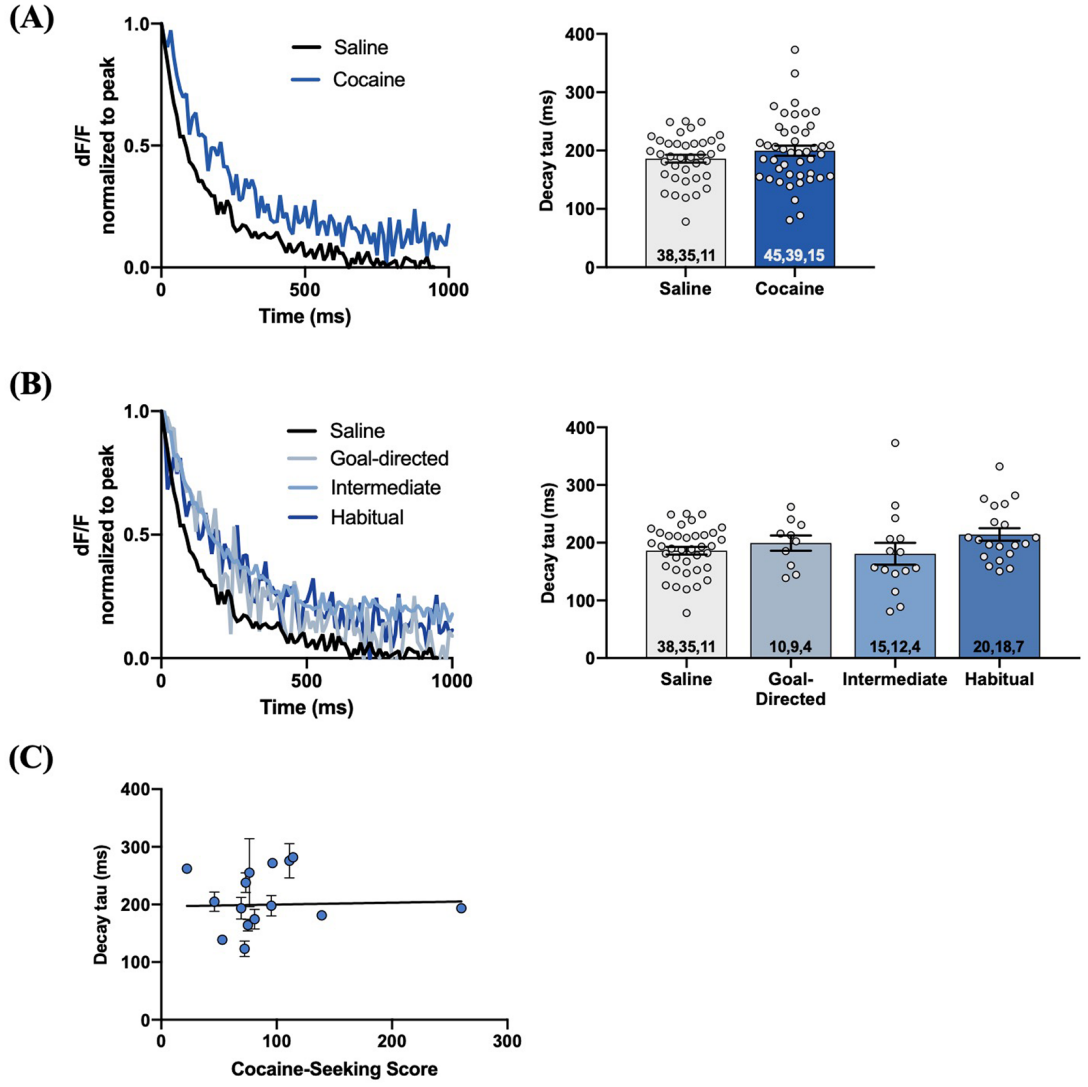
HFS-evoked iGluSnFr responses display no differences in glutamate dynamics in the DLS of cocaine-experienced animals and saline controls. **(A)** Representative peak-normalized iGluSnFr response decay evoked by 1 mA HFS (10 Hz, 1 s) in slices from drug-naïve control and cocaine-experienced animals. Decay tau quantified in the corresponding bar graph (unpaired *t*-test, *p* > 0.05). **(B)** Representative peak-normalised, HFS-evoked iGluSnFr response decay of goal-directed, intermediate, and habitual classifications. Decay tau quantified in the corresponding bar graphs (one-way ANOVA with Dunnet’s *post hoc* test, *p* > 0.05). **(C)** Relationship between cocaine-seeking score and decay tau of evoked iGluSnFr responses (Spearman correlation, *p* > 0.05), data fit with a linear regression line of best fit). iGluSnFr responses were evoked with 10 Hz HFS stimulation in the DLS of acute brain slices. Bar graphs are expressed as mean ± SEM. Sample sizes for each group **(A,B)** are denoted in each corresponding bar as: number of responses, number of slices, number of animals.

We examined iGluSnFr response decay kinetics to determine the rate of regional glutamate clearance following 1 mA double-pulse stimulation. The decay tau of evoked iGluSnFr responses was not significantly different between yoked-saline (143.8 ± 3.3 ms) and cocaine-experienced rats (140 ± 3.4 ms; *unpaired t-test, t = 0.69, p = 0.49*; Figure 6A). Analysis of the decay tau across the cocaine-experienced rats classified as goal-directed (138.7 ± 6.5 ms), intermediate (129.4 ± 5.3 ms), and habitual (150.2 ± 5.5 ms) and yoked-saline controls (143.8 ± 3.3 ms) did reveal an overall significant effect (*one-way ANOVA: F_(3, 155)_ = 2.6, p = 0.05*; Figure 6B). *Post hoc* analyses, however, failed to identify significant differences in decay tau between yoked-saline controls and each behavioral classification of cocaine-experienced rats (*Dunnett’s multiple comparisons adjusted p values*; *goal-directed: p = 0.82, intermediate: p = 0.10, habitual: p = 0.65*). Moreover, there was no significant correlation between cocaine-seeking score and decay tau of evoked iGluSnFr responses (*Spearman r = 0.05, p = 0.87;* Figure 6C). We observed similar results when iGluSnFr responses were evoked with 1 mA-HFS (0.1 ms pulses, 10 Hz for 1 s). The decay tau of HFS-evoked iGluSnFr responses did not significantly differ between yoked-saline (186.0 ± 6.6 ms) and cocaine-experienced rats (199.8 ± 8.6 ms; *unpaired t-test, t = 1.23, p = 0.22*; Figure 7A). Furthermore, there were no significant differences in decay tau across rats classified as goal-directed (199.4 ± 13.1 ms), intermediate (180.7 ± 19.0 ms), or habitual (214.2 ± 10.9 ms) in their cocaine seeking, both relative to each other and compared to yoked-saline controls (*Kruskall-Wallis statistic = 5.13, p = 0.16*; Figure 7B). Lastly, there was no significant correlation between decay tau and cocaine-seeking score (*Spearman r = 0.23, p = 0.40;* Figure 7C). Taken together, these results suggest that glutamate clearance in the DLS is unaffected by a history of cocaine self-administration as well as the development of habitual control over cocaine-seeking behavior.

**Figure 8:**
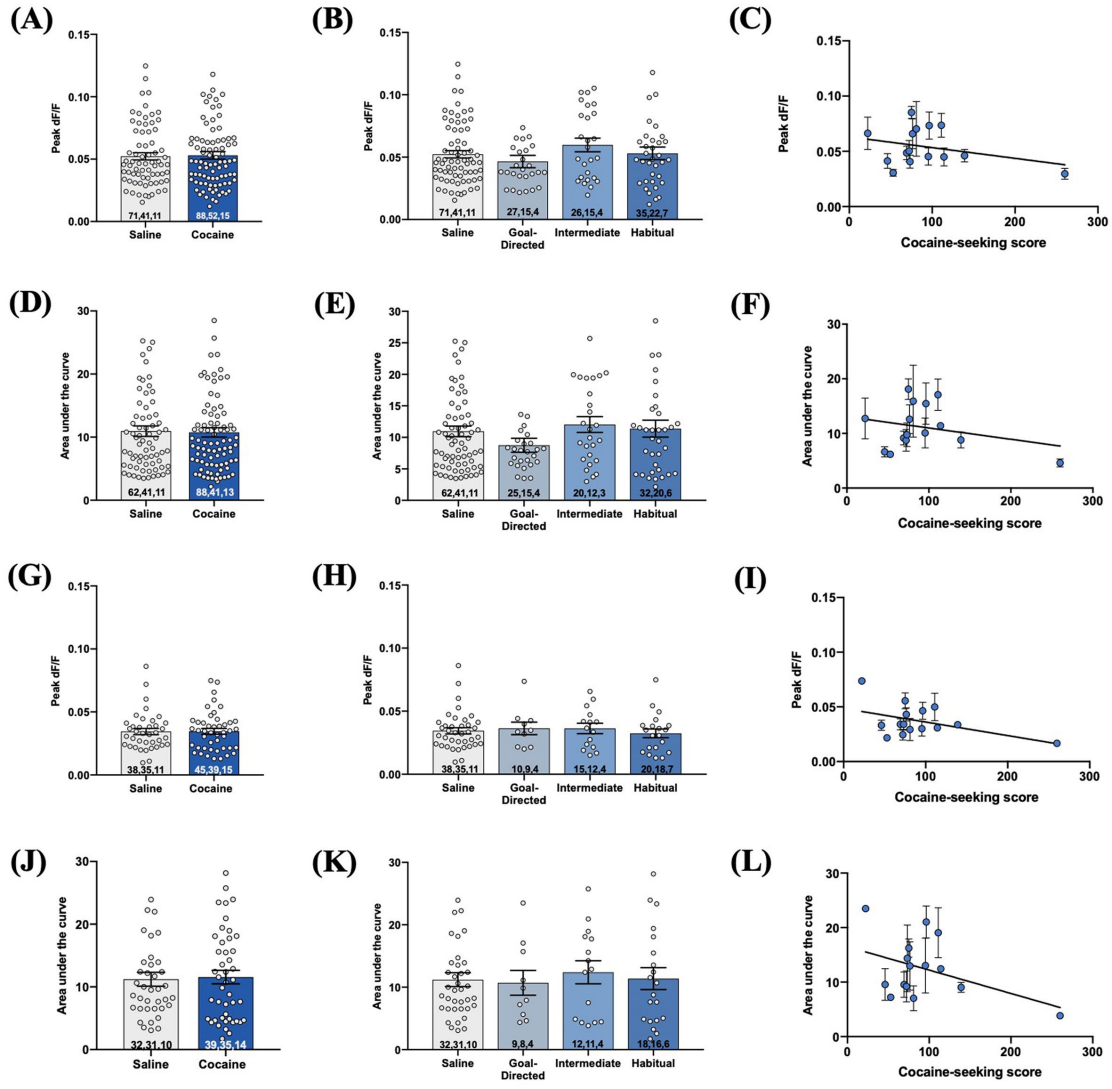
Evoked iGluSnFr responses in acute brain slices obtained from cocaine-experienced animals display no differences in synaptically-released glutamate in the DLS. iGluSnFr responses were evoked with either 1 mA double-pulse stimulation **(A-F)** or high-frequency stimulation **(G-L)**. **(A,G)** Peak signal (dF/F) of iGluSnFr responses in the DLS of yoked saline and cocaine- experienced animals (Mann-Whitney test, *p* > 0.05) and **(B,H)** across behavioral classification groups (Kruskall-Wallis test, *p* > 0.05). **(C,I)** Relationship between cocaine-seeking score and peak dF/F (Spearman correlation, *p* > 0.05). AUC of evoked iGluSnFr responses in the DLS of **(D,J)** yoked saline and cocaine-experienced animals (Mann-Whitney test, *p* > 0.05) and **(E,K)** across cocaine-seeking behavioral classification groups (Kruskall-Wallis test, *p* > 0.05). **(F,L)** Relationship between cocaine-seeking score and AUC (Spearman correlation, *p* > 0.05). iGluSnFr responses were evoked with either 1 mA double-pulse stimulation or 1 mA HFS (10 Hz, 1 s). Bar graphs expressed as mean ± SEM. Sample sizes for each group are denoted in each corresponding bar as: number of averaged responses, number of slices, number of animals.

As iGluSnFr provides the means to assess aspects of synaptic glutamate release (Marvin et al., 2013; Parsons et al., 2016; Koch et al., 2018), we also investigated these metrics in the DLS of cocaine-experienced rats. We first assessed these measures in iGluSnFr responses evoked with local 1 mA double pulse stimulation. There was no significant difference in the peak signal of evoked responses between cocaine-experienced and yoked saline rats (saline: 0.052 ± 0.003dF/F; cocaine: 0.053 ± 0.003dF/F; *Mann–Whitney U = 3,101, p = 0.94*; Figure 8A). Similarly, the peak signal was unchanged across goal-directed (0.046 ± 0.005 dF/F), intermediate (0.06 ± 0.005 dF/F), and habitual (0.05 ± 0.005 dF/F) cocaine-seeking rats and yoked-saline controls (*Kruskall-Wallis statistic = 3.46, p = 0.33*; Figure 8B). Furthermore, peak signal and cocaine-seeking score were not significantly correlated (*Spearman r = 0.01, p = 0.98*, Figure 8C). Next, there was no significant difference in the AUC of double-pulse-evoked responses between cocaine-experienced (10.76 ± 0.74) and yoked-saline (10.95 ± 0.84) rats (*Mann–Whitney U = 3,100, p = 0.93*; Figure 8D), as well as across behavioral classification groups (goal-directed: 8.7 ± 1.12; intermediate: 12.03 ± 1.25; habitual: 11.38 ± 1.35) and controls (*Kruskall-Wallis statistic = 4.25, p = 0.24*; Figure 8E). Finally, there was no significant correlation between AUC and cocaine-seeking score (*Spearman r = 0.12, p = 0.68*, Figure 8F). Similar results were observed when iGluSnFr responses were evoked with 1 mA-HFS. There were no significant differences in peak signal between cocaine-experienced (0.03 ± 0.002 dF/F) and yoked-saline (0.03 ± 0.002 dF/F) rats (*Mann Whitney U = 854.5, p = 0.99*, Figure 8G), as well as across behavioral classification groups (goal-directed: 0.04 ± 0.005 dF/F; intermediate: 0.04 ± 0.004 dF/F; habitual: 0.03 ± 0.003 dF/F) and yoked-saline controls (*Kruskall-Wallis statistic = 0.89, p = 0.83*, Figure 8H). In addition, there was no significant correlation between HFS-evoked peak signal and cocaine-seeking score (*Spearman r = −0.175, p = 0.53*, Figure 8I). Finally, there were no significant differences in the AUC between cocaine-experienced (11.57 ± 1.07) and yoked-saline (11.21 ± 1.1) rats (*Mann Whitney U = 840, p = 0.89*, Figure 8J), as well as across behavioral classification groups (goal-directed: 10.70 ± 1.98; intermediate: 12.40 ± 1.85; habitual: 11.37 ± 1.75) and yoked-saline controls (*Kruskall-Wallis statistic = 0.34, p = 0.95*, Figure 8K). Lastly, there was no significant correlation between the AUC of HFS-evoked iGluSnFr responses and cocaine-seeking score (*Spearman r = −0.17, p = 0.54*, Figure 8L). Taken together, these data suggest that iGluSnFr metrics of glutamate release in the DLS are unaltered in cocaine-experienced rats, regardless of the mode of control over their cocaine-seeking behavior.

### 3.4. GLT-1 protein expression in the DLS is unaltered following chained cocaine self-administration

**Figure 9:**
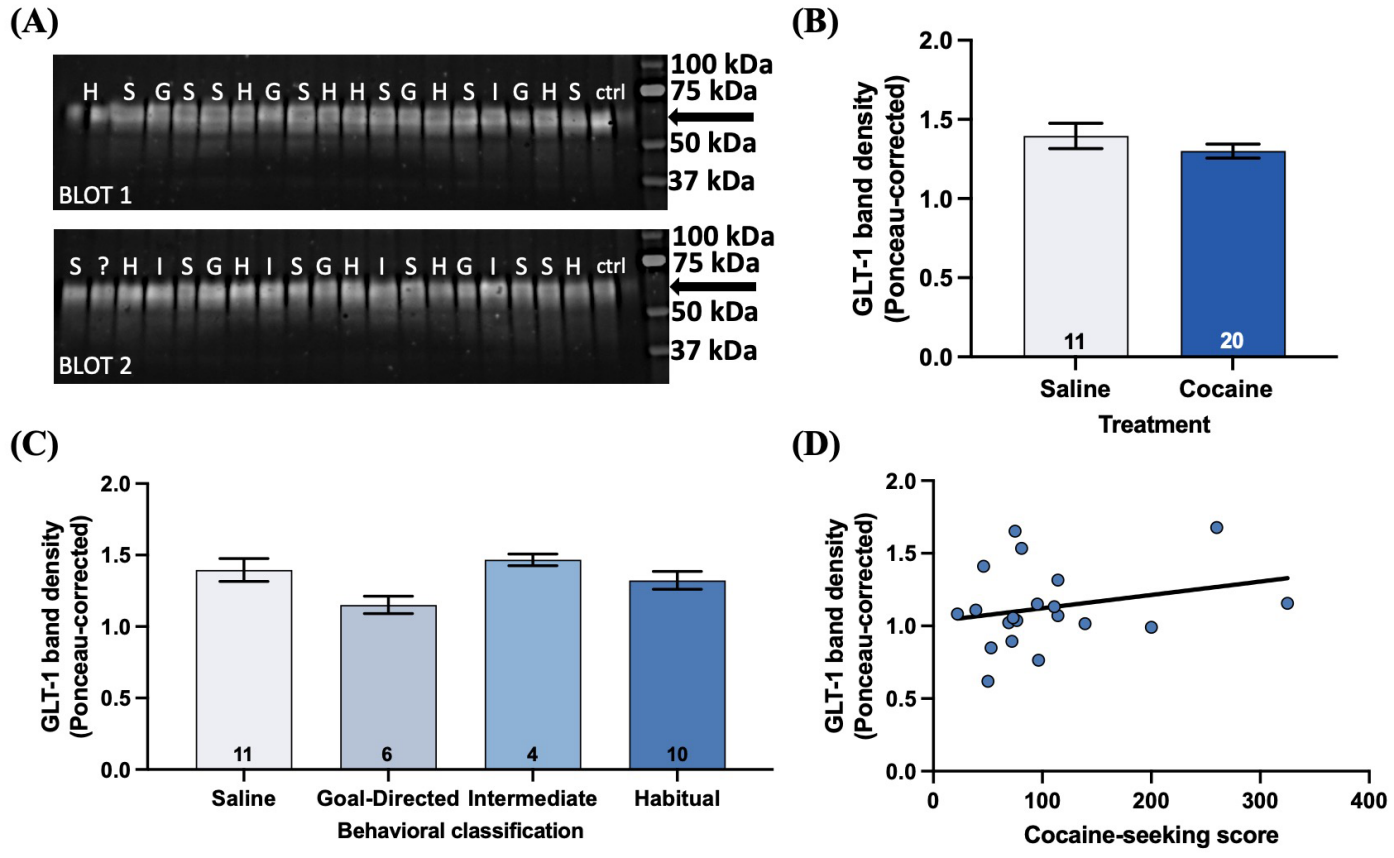
GLT-1 expression in the DLS is unaltered by cocaine-experience or the mode of behavioral control over cocaine-seeking. **(A)** Illustrative western blots from saline (S), goal-directed (G), intermediate (I), and habitual (H) groups for GLT-1 protein obtained from micro-punches of the DLS. The GLT-1 band resolved at ~62 kDa (arrow). “?” indicates sample from an animal who failed to respond on either seeking test and therefore could not be behaviorally classified. “ctrl” indicates control lanes. **(B)** GLT-1 band density in saline and cocaine-treated animals (unpaired *t*-test, *p* > 0.05). **(C)** Ponceau-corrected GLT-1 band density across goal-directed, intermediate, and habitual classification groups (one-way ANOVA with Dunnet’s *post hoc* test, *p* > 0.05). **(D)** Relationship between cocaine-seeking score and GLT-1 band density (Spearman correlation, *p* > 0.05). Data fit with a linear regression line of best fit. Bar graphs expressed as mean ± SEM. Sample sizes for each group **(B,C)** are denoted in each corresponding bar as number of animals.

Given the extensive literature reporting decreased GLT-1 expression in the accumbens of rats with a history of cocaine self-administration and withdrawal (Kalivas, 2009; Niedzielska-Andres et al., 2021) and one report of decreased expression in the DLS (Ducret et al., 2016), we investigated GLT-1 protein expression via western blotting of the DLS of cocaine-experienced rats and across cocaine-seeking behavioral classifications (Figure 9A). There was no significant difference in ponceau-corrected GLT-1 band intensity in cocaine-experienced (1.30 ± 0.04) rats in comparison to yoked-saline controls (1.40 ± 0.08; *unpaired t-test, t = 1.14, p = 0.26*; Figure 9B). Similarly, sub-group analysis of the cocaine-experienced rats showed that there was no difference in GLT-1 expression in the DLS of rats classified as goal-directed (1.15 ± 0.06), intermediate (1.47 ± 0.04), or habitual (1.32 ± 0.06) in their cocaine-seeking relative to each other and the yoked-saline controls (*one-way ANOVA: F_(3, 27)_ = 2.39, p = 0.09*; Figure 9C). Finally, there was no significant correlation between cocaine-seeking score and GLT-1 expression in the DLS (*Spearman r = 0.21*, *p = 0.37*; Figure 9D). These results suggest that extended cocaine self-administration, regardless of apparent goal-directed, intermediate, or habitual control over cocaine-seeking behavior, is not associated with altered GLT-1 protein expression in the DLS.

## 4. Discussion

The transition between recreational drug use and addiction is considered to arise from the shift between goal-directed and habitual control over drug behaviors. While the development of habitual appetitive and motor skill behaviors has been shown to be mediated by potentiated glutamatergic signaling in the DLS (Yin et al., 2009; Goodman et al., 2017), the state of the DLS glutamate system in the context of habitual drug behaviors has remained undefined. Therefore, this study profiled the state of the DLS glutamate system across rats whose cocaine-seeking behavior was classified as goal-directed, intermediate, and habitual based on sensitivity to outcome omission. Overall, we found that glutamate clearance and release dynamics and GLT-1 protein expression in the DLS are largely unchanged across cocaine-experienced rats, both relative to the apparent nature of the behavioral control over the cocaine-seeking behavior and relative to yoked-saline controls.

The present study capitalized on a previously published behavioral paradigm that enabled the classification of cocaine-seeking as either goal-directed or habitual (Zapata et al., 2010). Similar to Zapata et al. (2010), training on this paradigm reliably produced goal-directed and habitual cocaine-seeking rats. However, we also observed a subset of a rats whose cocaine-seeking scores fell between the goal-directed and habitual categories, which we termed “intermediate.” An intermediate cocaine-seeking score likely represents a rat whose cocaine-seeking behavior was transitioning between goal-directed and habitual control at the time of testing or were, perhaps, more flexible in their behavioral control. Indeed, Zapata et al. (2010) demonstrated that rats with goal-directed cocaine-seeking behavior transitioned to habitual cocaine-seeking with extended training. Next, rats in the present study, as well as in Zapata et al. (2010), demonstrated a wide range of sensitivity of their cocaine-seeking behavior to outcome devaluation, despite being given the same daily access to cocaine self-administration sessions. Whether this wide range in cocaine-seeking scores is due to individual differences in learning strategy or vulnerability to addiction (Belin et al., 2009) remains an interesting avenue of future study. Finally, we observed some minor differences in lever press behavior between iGluSnFr-transfected rats and non-transfected rats during self-administration training. Namely, iGluSnFr-transfected rats made more taking-lever presses than non-transfected rats during the initial FR1 and revaluation FR1 training sessions and also made more seeking-lever presses during chain 4 training. These training differences may have stemmed from the stereotaxic surgery and/or DLS infusion itself that the iGluSnFr group received, as stress, for example, has been shown to influence instrumental responding (Schwabe and Wolf, 2009; Smeets et al., 2019). In addition, iGluSnFr itself introduces an artificial, competitive binding site for extracellular glutamate (Marvin et al., 2018; Armbruster et al., 2020), which needs to be acknowledged when combined with behavioral studies. Indeed, computational analyses suggest that iGluSnFr expression on astrocytes decreases the rate of glutamate uptake into astrocytes, which can result in an increased time course and concentration of extracellular glutamate (Armbruster et al., 2020). It is, therefore, plausible that the iGluSnFr expression in the DLS of rats in the present study may have altered glutamate dynamics within the DLS and affected their instrumental training, but further study is needed. Nevertheless, we found that none of these training differences were related to the final cocaine-seeking score of rats in either group and, importantly, iGluSnFr transfection did not significantly affect the average cocaine-seeking score of rats. Moreover, as training on this behavioral paradigm reliably produced goal-directed and habitual cocaine-seeking rats, and it has previously been used to demonstrate that habitual cocaine-seeking is dependent on DLS activity (Zapata et al., 2010), we considered it to be an ample platform to assess the state of the DLS glutamate system in goal-directed and habitual cocaine-seeking rats.

We used whole-cell patch-clamp electrophysiology of DLS astrocytes in acute brain slices to assess glutamate dynamics in rats with a history of cocaine self-administration exhibiting goal-directed or habitual cocaine-seeking behavior. STCs evoked by single pulse stimulation displayed significantly slower rates of glutamate clearance in slices obtained from cocaine-experienced rats, as observed by an increase in the decay tau and half-width values. Interestingly, when decay tau and half-width values were compared between saline and either the goal-directed or habitual groups, there were no significant differences, nor was there any relationship between cocaine-seeking scores and decay tau values. Single-pulse evoked STCs, therefore, suggest that cocaine-experience, independent of goal-directed or habitual control over cocaine-seeking behavior, is associated with a slower rate of glutamate clearance in the DLS. In contrast, analysis of STC recordings evoked by HFS indicate no significant differences in decay tau values between yoked-saline controls and rats with a history of cocaine self-administration. Similarly, there were no differences observed in accordance with any of the behavioral classification groups, nor a significant relationship between cocaine-seeking score and HFS decay tau. As HFS does not overwhelm astrocyte transporters (Diamond and Jahr, 2000) and has been shown to reveal impairments in glutamate clearance that are not apparent or well-defined under low frequency conditions (Tanaka et al., 2013; Umpierre et al., 2019), the lack of a significant difference in HFS decay tau values seemingly contradicts our findings from single-pulse STCs. However, given the small, albeit statistically significant, difference in single-pulse decay tau values between saline (11.9 ± 0.3 ms) and cocaine-experienced (12.9 ± 0.3 ms) rats, the lack of a significant difference in HFS decay tau likely reflects that there are no biologically relevant changes. Therefore, taken together, our results from evoked STCs suggest that there are seemingly negligible changes to the rate of glutamate clearance in the DLS of cocaine-experienced rats, regardless of whether such cocaine-seeking behavior is classified as goal-directed or habitual. It is important to acknowledge that STC recordings may reflect the intrinsic properties of the glutamate transporter, not necessarily the rate of glutamate clearance (Wadiche et al., 2006), and do not provide insight to other cellular events occurring at the synapse. Alternative glutamate homeostatic mechanisms of the tripartite synapse including GLAST expression, cysteine-glutamate exchanger, astrocytic ensheathment of neurons, and neuronal function may act in a compensatory manner (Mahmoud et al., 2019), resulting in no detectable changes in STC recordings. Future studies of these aspects of glutamate dynamics are needed to fully characterize synapse properties in the DLS of cocaine-experienced rats classified as goal-directed or habitual in their cocaine-seeking behavior.

We also expressed iGluSnFr on astrocytes and astrocyte processes in the DLS to visualize real-time extracellular glutamate dynamics at a regional level. The rate of glutamate clearance, as reflected by the decay tau of evoked iGluSnFr signals, did not differ across goal-directed, intermediate, and habitual cocaine-seeking rats and yoked-saline controls. In addition, no group differences were observed in the magnitude of evoked glutamate release and the total amount of glutamate sensed by iGluSnFr-expressing astrocytes, as reflected by the peak signal and area under the curve of evoked iGluSnFr signals, respectively. Notably, these results were observed in response to both double-pulse stimulation as well as HFS, suggesting that the glutamate clearance system is intact under low-stimulation conditions and in glutamate spillover conditions (Umpierre et al., 2019). Importantly, we verified that iGluSnFr imaging in our laboratory was functioning properly, as we demonstrated that evoked iGluSnFr responses were sensitive to a wide range of experimental manipulations (Parsons et al., 2016), including TTX-induced blockade of action-potential dependent glutamate release, blockade of glutamate transporters with TBOA, and an increased bath temperature. As such, the data suggest that there is little difference in extracellular glutamate dynamics in the DLS of cocaine-experienced rats, regardless of whether their cocaine-seeking behavior is characterized as habitual, intermediate, or goal-directed.

In line with the negligible differences in glutamate clearance observed by STCs and iGluSnFr assays, the level of GLT-1 protein expression in the DLS was also unchanged between cocaine-experienced rats, regardless of behavioral classification, and yoked-saline controls. This finding further builds the profile of an intact glutamate clearance system in the DLS of habitual cocaine-seeking rats, as GLT-1 is the predominant glutamate transporter and accounts for ~90% of glutamate clearance in the brain (Danbolt, 2001; Magi et al., 2019).

The present results are surprising, as enduring changes in the glutamate system have been observed in the NAc of cocaine-experienced rats (Kalivas, 2009; Niedzielska-Andres et al., 2021). Specifically, rats with a history of cocaine self-administration exhibit decreased GLT-1 expression and decreased uptake capacity of glutamate transporters in the NAc (Knackstedt et al., 2010; Sari et al., 2010; Reissner et al., 2012, 2014; Sondheimer and Knackstedt, 2012; Fischer-Smith et al., 2013; Trantham-Davidson et al., 2013; LaCrosse et al., 2017). In addition, the magnitude of synaptically-released glutamate in the NAc is also increased upon cue or cocaine-primed reinstatement (McFarland et al., 2003; Madayag et al., 2007; Li et al., 2010). Together, these alterations to presynaptic glutamate release and glutamate clearance largely contribute to the enduring vulnerability to relapse (Kalivas, 2009; Niedzielska-Andres et al., 2021). In comparing the present results to this literature, it is important to consider the methodologies used and how they provide different perspectives into the glutamate system. First, the assessments of glutamate clearance in the NAc were conducted with the tritiated glutamate uptake assay, which measures the total uptake capacity of glutamate transporters on the timescale of minutes (Knackstedt et al., 2010; Trantham-Davidson et al., 2013). This differs from the rapid clearance of glutamate that occurs on the millisecond time scale (Clements et al., 1992) that is detected through STC recordings (Diamond and Jahr, 2000; Diamond, 2005) and iGluSnFr imaging (Marvin et al., 2013; Parsons et al., 2016; Pinky et al., 2018). Similarly, seemingly contradictory results across these different assays of glutamate clearance have been observed in the dorsal striatum of mice with Huntington’s disease (HD). Here, HD mice exhibited decreased GLT-1 expression and decreased uptake capacity of glutamate transporters, but the rate of glutamate clearance was unaffected, and even increased, when measured with iGluSnFr imaging and STCs (Parsons et al., 2016; Raymond, 2017). Therefore, as these assays measure different dimensions of glutamate clearance, the present results should be viewed as providing a more comprehensive, rather than contradictory, view of glutamate dynamics in the cocaine-experienced striatum.

While less profiled than the NAc, changes in the DLS glutamate system have also been reported in the context of cocaine self-administration. That is, decreased GLT-1 protein expression was observed in rats given long (6 h), but not short (2 h), access to repeated cocaine self-administration (Ducret et al., 2016). Given that the short-access condition is similar to the 1–3 h timeframe of cocaine access used in the present study, differences in the glutamate system may emerge if rats are given longer daily access to cocaine self-administration. Indeed, GLT-1 expression in the NAc has been shown to be differentially affected by the length of daily cocaine access sessions as well as the length of cocaine withdrawal (Fischer-Smith et al., 2013; Ducret et al., 2016). Further, rats in Ducret et al. (2016) were sacrificed after numerous tests, including punishment-induced abstinence, which may have contributed to changes in GLT-1 expression in the DLS. It has also been reported that rats withdrawn from cocaine self-administration for 24 h exhibit increased extracellular glutamate levels in the DLS in response to an acute cocaine challenge (Gabriele et al., 2012). Notably, our rats were not challenged prior to sacrifice, which occurred 1–4 days after the final drug-seeking test (and 2–5 days after the last day of cocaine self-administration). Thus, although these two prior studies have reported changes in aspects of glutamate signaling in the DLS in rats with a history of cocaine self-administration, the present data suggest that those changes may be unique to the behavioral history of the animals and/or acute drug challenge. However, the present results suggest that a history of cocaine-seeking and taking, regardless of whether the learned cocaine-seeking behavior is apparently goal-directed or habitual, is not associated with changes in glutamate clearance or release nor changes in GLT-1 expression in the DLS.

The present results may have implications for the use of treatments aimed at restoring glutamate homeostasis in individuals with cocaine use disorder. To date, there are no FDA-approved medications for the treatment of cocaine use disorder (National Institute on Drug Abuse, 2021). However, given the large body of work detailing the decreased expression of GLT-1 and xCT (the catalytic subunit of the cysteine-glutamate exchanger xC-) in the NAc (Niedzielska-Andres et al., 2021), agents that restore expression and function of these proteins, such as N-acetylcysteine and Ceftriaxone, have received increasing therapeutic attention (Nocito Echevarria et al., 2017; Spencer and Kalivas, 2017). In light of the present results, however, the effect of these systemically-administered glutamate-targeted interventions on the DLS glutamate system and habitual responding need to be considered. Given that the present results suggest that glutamate release, clearance, and GLT-1 expression are not altered in the DLS of cocaine-experienced rats, administering agents that increase glutamate clearance and basal levels of extracellular glutamate may adversely impact instrumental responding. As such, further study is needed to explore the effect of NAC and Ceftriaxone on habitual responding for cocaine, as well as the underlying glutamate dynamics in the DLS, in order to further elucidate their efficacy in treating the striatally-based behavioral aspects of cocaine use disorder. Importantly, as the present study was conducted only in male rats, assessing these measures in female rats is needed in order to obtain a comprehensive understanding of cocaine abuse disorder and to adequately inform the development of treatment strategies.

Overall, we found that the dynamics of glutamate release and clearance as well as GLT-1 protein expression in the DLS are largely unchanged across rats with goal-directed, intermediate, and habitual cocaine-seeking behavior, as well as in comparison to yoked saline controls. These results provide a more comprehensive, rather than contrasting, profile of glutamate dynamics in the cocaine-experienced striatum and spark a deeper conversation into the state of the striatal glutamate system in the context of cocaine use. Namely, it is imperative to consider both the specific glutamate assay and cocaine paradigm employed when conducting and comparing studies on rats with a history of cocaine self-administration and different addiction-related phenotypes. Moreover, this study urges that more comprehensive profiles of the DLS glutamate system and habitual cocaine-related behavior are not only developed, but considered, as glutamate-targeted therapies for cocaine use disorder advance to clinical application.

## Data availability statement

The raw data supporting the conclusions of this article will be made available by the authors, without undue reservation.

## Author contributions

KK and KW conceived the project. KK, KW, DG, and KV designed the experiments. DG and KV carried out the iGluSnFr and STC experiments, respectively, and collected the data. DG and MT established the cocaine self-administration paradigm and stereotaxic surgical methods. MT, DG, KV, and KK carried out the cocaine self-administration training. MT conducted the stereotaxic surgeries. PW assisted with electrophysiology experiments and data analysis. DG and KV analyzed the data and wrote the final manuscript. All authors contributed to the article and approved the submitted version.

## Funding

This work was supported by the National Institutes of Health (R21DA0466000), University of Utah Skaggs Graduate Research Fellowship, Roy Kuramoto Award, and University of Utah Neuroimmunology Training Grant (T32NS115664).

## Conflict of interest

The authors declare that the research was conducted in the absence of any commercial or financial relationships that could be construed as a potential conflict of interest.

## Publisher’s note

All claims expressed in this article are solely those of the authors and do not necessarily represent those of their affiliated organizations, or those of the publisher, the editors and the reviewers. Any product that may be evaluated in this article, or claim that may be made by its manufacturer, is not guaranteed or endorsed by the publisher.
