# Supplementary figures and images for: Glutamate dynamics in the dorsolateral striatum of rats with goal-directed and habitual cocaine-seeking behavior
Source: Front Mol Neurosci. 2023 May 11;16:1160157. doi: 10.3389/fnmol.2023.1160157 (PMC10213946; doi:10.3389/fnmol.2023.1160157)

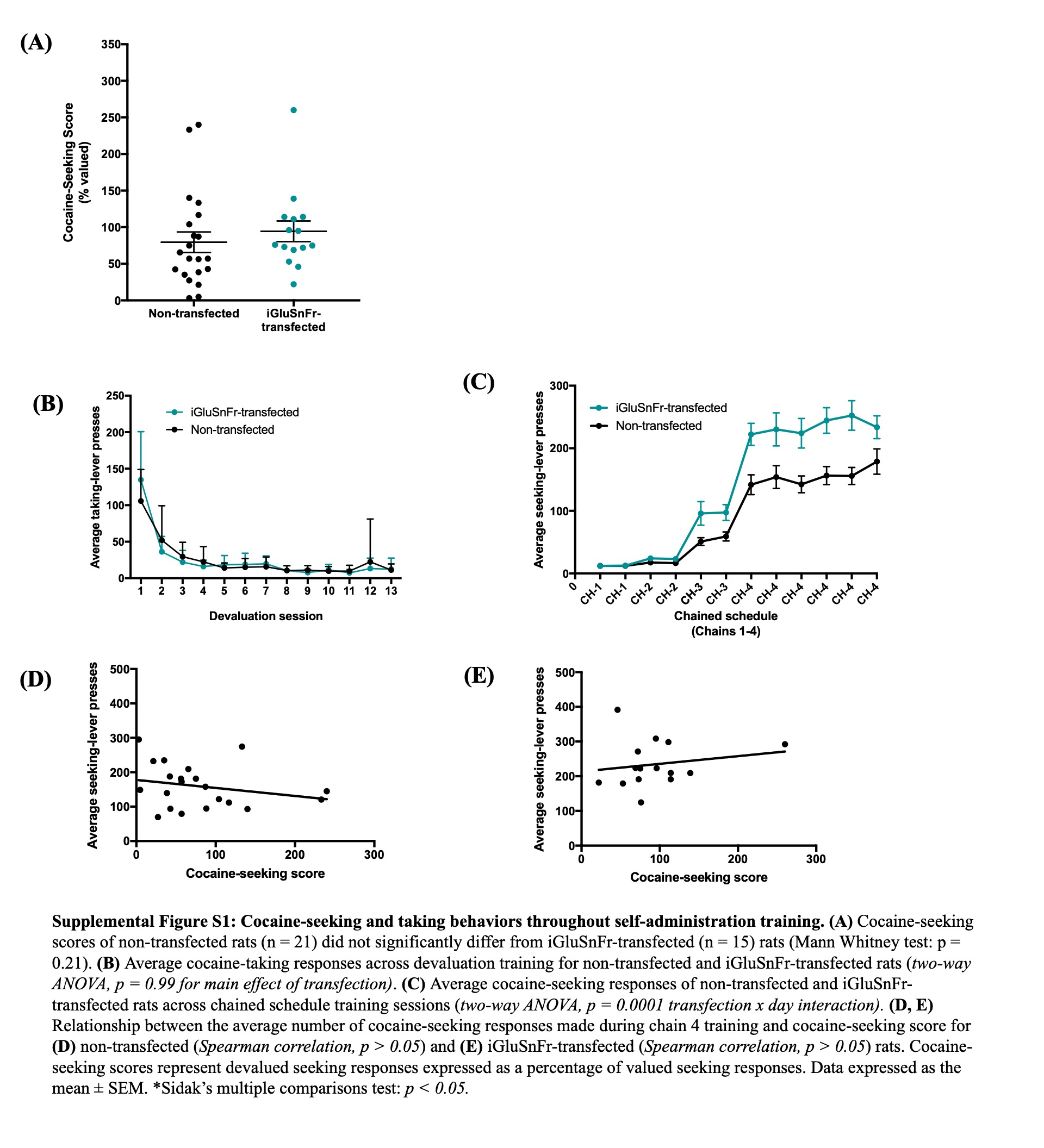

Supplement: Supplementary file 1 [file Image_1.jpeg]

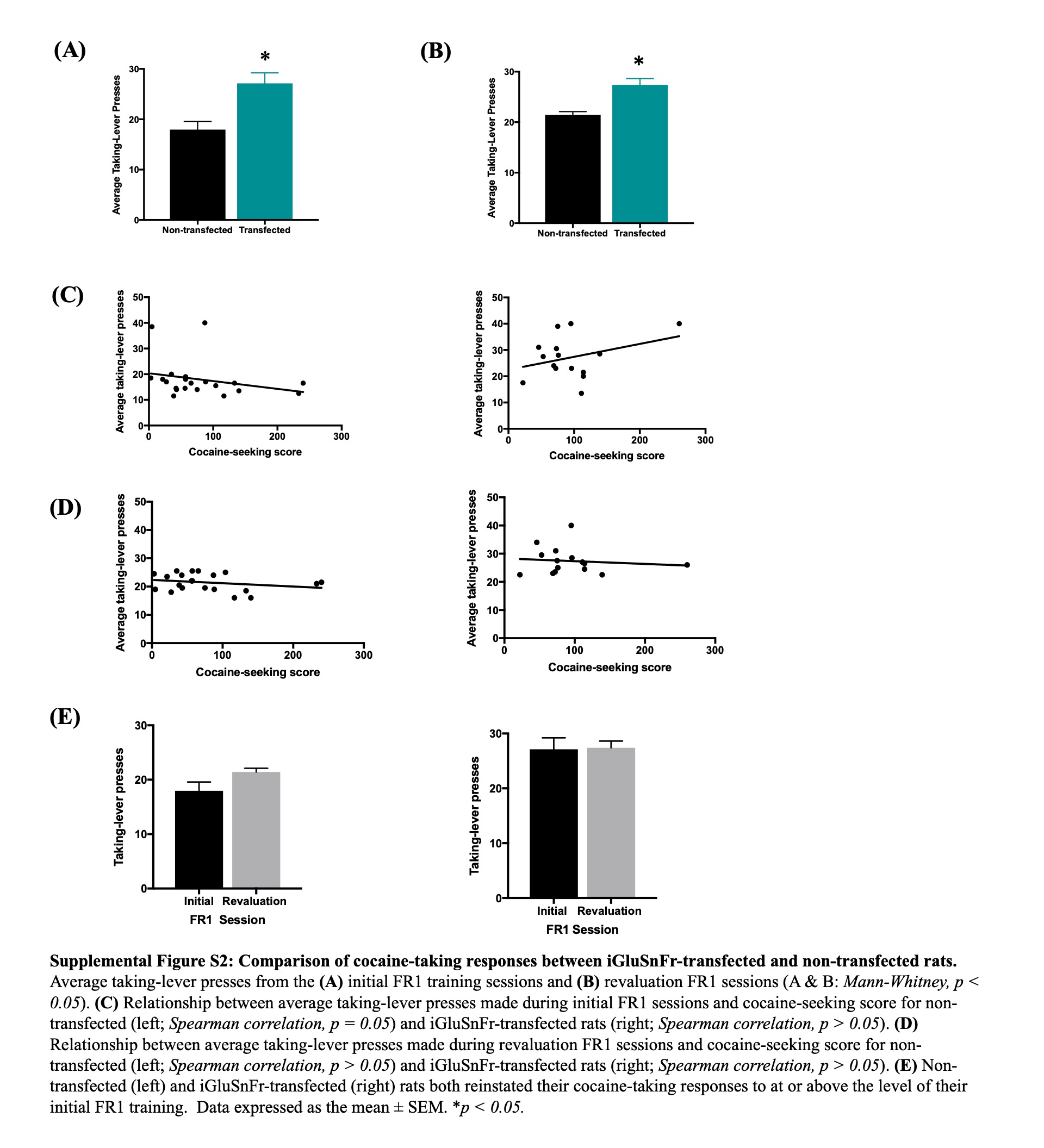

Supplement: Supplementary file 2 [file Image_2.JPEG]

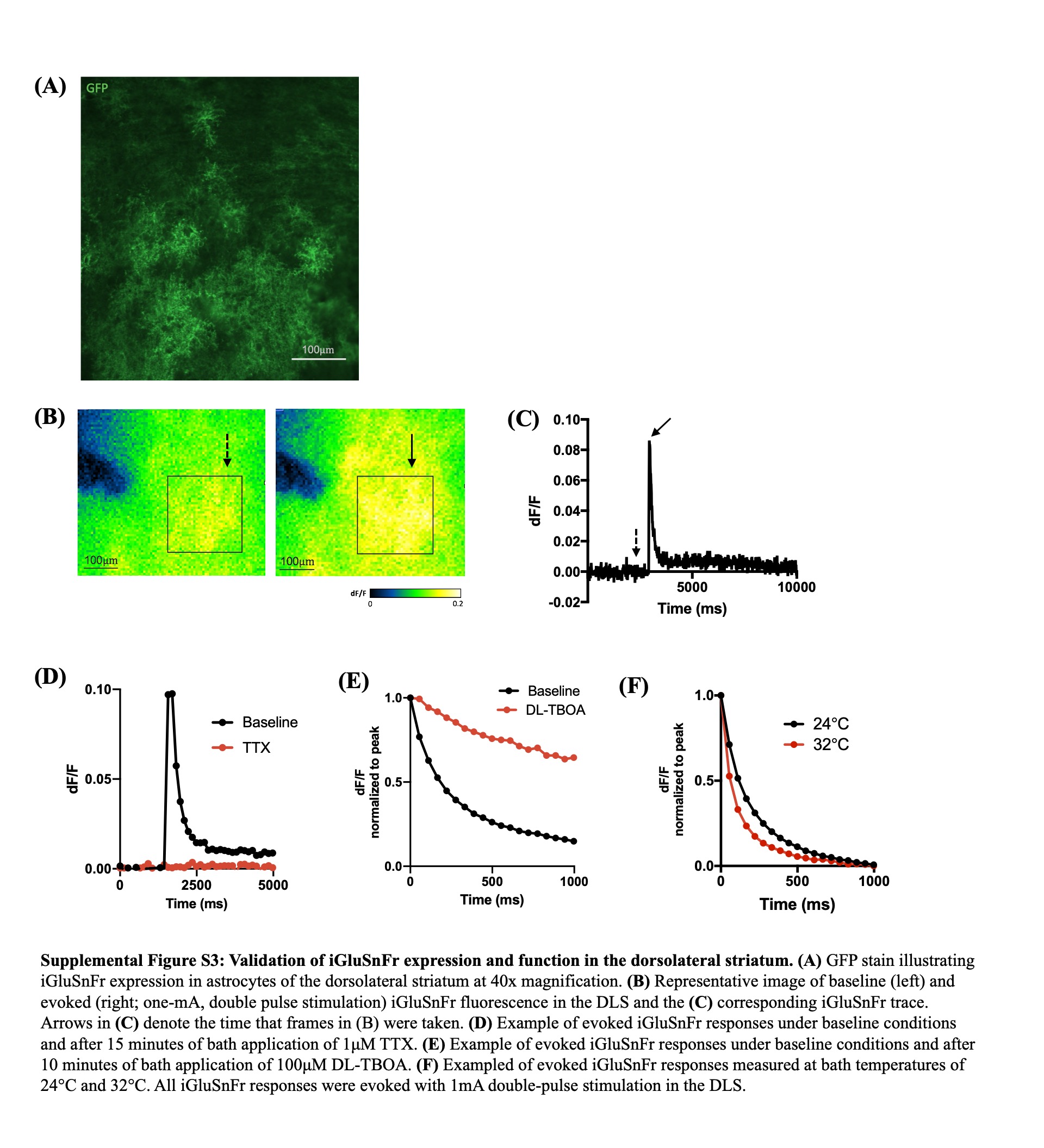

Supplement: Supplementary file 3 [file Image_3.jpeg]
